# Current status of clinical trials assessing mesenchymal stem cell therapy for graft versus host disease: a systematic review

**DOI:** 10.1186/s13287-022-02751-0

**Published:** 2022-03-04

**Authors:** Ying Li, Jie Hao, Zheng Hu, Yong-Guang Yang, Qi Zhou, Liguang Sun, Jun Wu

**Affiliations:** 1 Key Laboratory of Organ Regeneration and Transplantation of Ministry of Education, The First Hospital of Jilin University, Changchun, 130061 China; 2 National-Local Joint Engineering Laboratory of Animal Models for Human Diseases, Changchun, 130061 China; 3 Department of Gastroenterology, The First Hospital, Jilin University, Changchun, 130021 China; 4 State Key Laboratory of Stem Cell and Reproductive Biology, Institute of Zoology, Chinese Academy of Sciences, Beijing, 100101 China; 5 Institute for Stem Cell and Regeneration, Chinese Academy of Sciences, Beijing, 100101 China; 6 National Stem Cell Resource Center, Chinese Academy of Sciences, Beijing, 100101 China; 7 International Center of Future Science, Jilin University, Changchun, 130021 China; 8 University of Chinese Academy of Sciences, Beijing, 100049 China

**Keywords:** Graft-versus-host disease, Mesenchymal stem cells, Clinical trials

## Abstract

**Background:**

Graft-versus-host disease (GVHD) is a common fatal complication of hematopoietic stem cell transplantation (HSCT), where steroids are used as a treatment option. However, there are currently no second-line treatments for patients that develop steroid-resistance (SR). Mesenchymal stem cells (MSCs) have immunomodulatory functions and can exert immunosuppressive effects on the inflammatory microenvironment. A large number of in vitro experiments have confirmed that MSCs can significantly inhibit the proliferation or activation of innate and adaptive immune cells. In a mouse model of GVHD, MSCs improved weight loss and increased survival rate. Therefore, there is great promise for the clinical translation of MSCs for the prevention or treatment of GVHD, and several clinical trials have already been conducted to date.

**Main body:**

In this study, we searched multiple databases and found 79 clinical trials involving the use of MSCs to prevent or treat GVHD and summarized the characteristics of these clinical trials, including study design, phase, status, and locations. We analyzed the results of these clinical trials, including the response and survival rates, to enable researchers to obtain a comprehensive understanding of the field’s progress, challenges, limitations, and future development trends. Additionally, factors that might result in inconsistencies in clinical trial results were discussed.

**Conclusion:**

In this study, we attempted to analyze the clinical trials for MSCs in GVHD, identify the most suitable group of patients for MSC therapy, and provide a new perspective for the design of such trials in the future.

**Supplementary Information:**

The online version contains supplementary material available at 10.1186/s13287-022-02751-0.

## Introduction

Hematopoietic stem cell transplantation (HSCT) is the best treatment modality for patients with hematological diseases that are incurable by conventional therapy [1–3]. HSCT procedures continue to increase annually, with more than 22,000 procedures performed in the USA in 2019 according to the statistics from the Center for International Blood and Marrow Transplant Research (CIBMTR), including more than 14,000 cases of autologous HSCT and more than 8000 cases of allogeneic HSCT [4]. In the same year, nearly 10,000 cases of allogeneic HSCT were accomplished in China [5]. During HSCT, transplanted immunocompetent lymphocytes can extensively recognize recipient antigens. These immune responses develop in a proinflammatory microenvironment resulting in clinical manifestations of GVHD, including rashes, elevated serum bilirubin levels, and diarrhea [6, 7]. In HSCT performed on HLA-identical siblings, acute GVHD (aGVHD) incidence is approximately 40%, reaching 80% for HLA-mismatched unrelated donors [8]. Acute GVHD is classified into four categories based on the severity of its manifestation: I (mild), II (moderate), III (severe), and IV (very severe) [9]. Furthermore, 35–50% of patients undergoing HSCT develop chronic GVHD (cGVHD) [10]. In attempts to control GVHD, various immunosuppressants have been used in the clinical prophylaxis and treatment of GVHD [11]. A steroid regimen remains the standard treatment, with less than 50% effectiveness [12] and no standard second-line treatment is currently available [13]. Patients with severe GVHD have dismal overall survival rates, with 25% for grade III and 5% for grade IV [14]. GVHD poses a growing threat to patient survival. Therefore, new effective treatment methods are warranted.

Mesenchymal stem cells (MSCs; also referred to as mesenchymal stromal cells) [15] are adherent fibroblast-like cells, capable of self-renewal and differentiation into chondrocytes, osteocytes, and adipocytes [16]. In terms of their phenotype, they are typically identified by their co-expression of CD29, CD44 [17, 18], CD73, CD90, CD105, and Sca-1 [19], and their non-expression of hematopoietic markers CD34, CD45, and CD11b [20]. Sufficiently high levels of proinflammatory cytokines can induce MSCs to exert immunosuppressive effects, making them suitable for controlling aberrant inflammatory responses [21, 22]. Bartholomew et al. [23] first reported that MSCs could prolong skin graft survival compared to the control group. Since then, many in vivo and in vitro experiments have demonstrated that MSCs have immunomodulatory capacities [24, 25], making them an ideal alternative to conventional immunosuppressants for GVHD. Mouse models have shown that injecting MSCs after bone marrow (BM) transplantation reduces the progression of GVHD [26–28]. Therefore, substantial clinical trials have been performed to evaluate the safety and efficacy of MSCs in steroid-resistance (SR) GVHD prevention or treatment.

## Overview of clinical trials

**Fig. 1:**
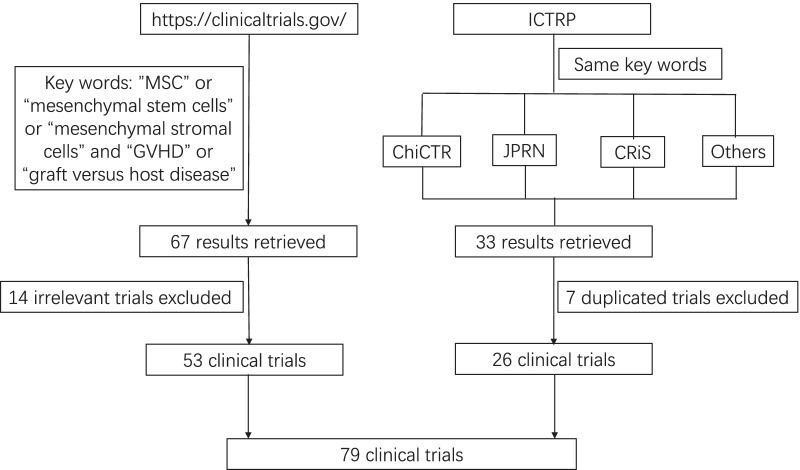
Flow chart of clinical trials involving MSC prevention or therapy for GVHD selected. Others represents Iranian Registry of Clinical Trials (IRCT), EU Clinical Trials Register, and Australian New Zealand Clinical Trials Registry (ANZCTR)

To comprehensively analyze clinical trials of MSCs for GVHD prevention or treatment, firstly, we searched the keywords "MSCs" OR “mesenchymal stem cells” OR “mesenchymal stromal cells” AND "GVHD" OR “graft versus host disease” on http://clinicaltrials.gov and obtained 67 results. After analyzing every retrieved result, 14 irrelevant clinical trials were excluded. Next, we searched the International Clinical Trials Registry Platform (ICTRP) search portal, the WHO's international clinical trial registration platform, with links to clinical trial registration platforms of different countries. Using the same keywords as above, we searched clinical trial registry platforms in each listed country or region, excluding 7 trials that had already been registered on http://clinicaltrials.gov, and found additional 26 trials (7 trials on Chinese Clinical Trial Registry (ChiCTR), 5 on Japan Primary Registries Network (JPRN), 1 on Clinical Research Information Service (CRiS), Republic of Korea, 1 on Iranian Registry of Clinical Trials (IRCT), 9 on EU Clinical Trials Register, and 3 on Australian New Zealand Clinical Trials Registry (ANZCTR)) (Additional file 1: Table S1). Consequently, 79 clinical trials were selected (Fig. 1) with a total of 4710 subjects enrolled (Additional file 2: Table S2). We numbered all the retrieved clinical trials (Additional file 1: Table S1), when a clinical trial is mentioned again below, we will use its number instead of its complete NCT or ID number. Except for clinical trials No.50 and No.51, with expanded access study type, and trial No.57, with observational study type, all others were interventional. Except for trial No.12, terminated after including one male patient, other clinical trials included both male and female patients. We analyzed the study design, phase, locations, and other characteristics of the 79 clinical trials.

**Fig. 2:**
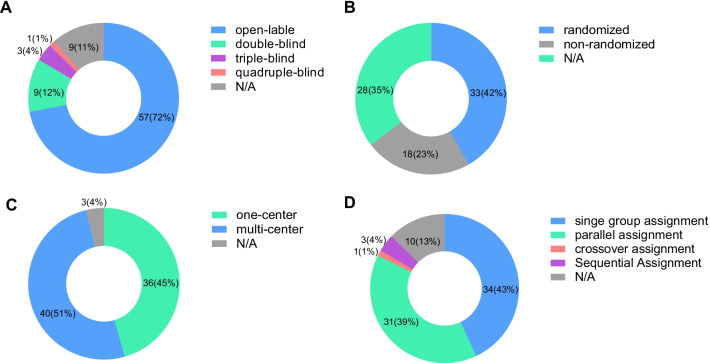
Study design of clinical trials involving MSC prevention or therapy for GVHD. **A** Masking of clinical trials, **B** allocation of clinical trials, **C** number of centers, **D** intervention model of clinical trials. *N/A*: not reported

Fifty-seven clinical trials were open-label; 9 were double-blinded for the participants and care providers; 3 were triple-blinded for the participants, care providers, and investigators; 1 was quadruple-blinded for the participants, care providers, investigators, and outcome assessors. Thirty-three clinical trials were randomized, 18 were non-randomized. 36 clinical trials were carried out in one center, and 40 were multi-center studies. The main intervention models were single-group assignment and parallel assignment. One study used a crossover assignment, while 3 used sequential assignments (Fig. 2).

**Fig. 3:**
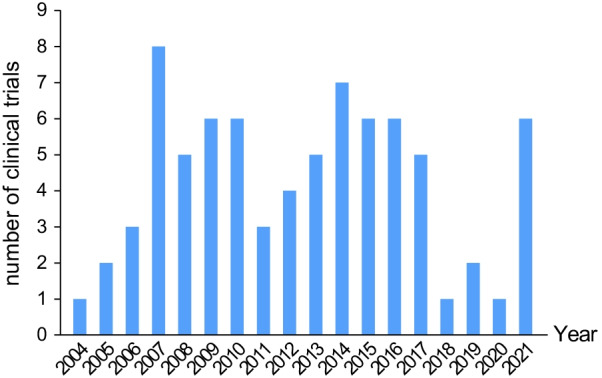
Number of clinical trials involving MSC prevention or therapy for GVHD initiated in each year

**Fig. 4:**
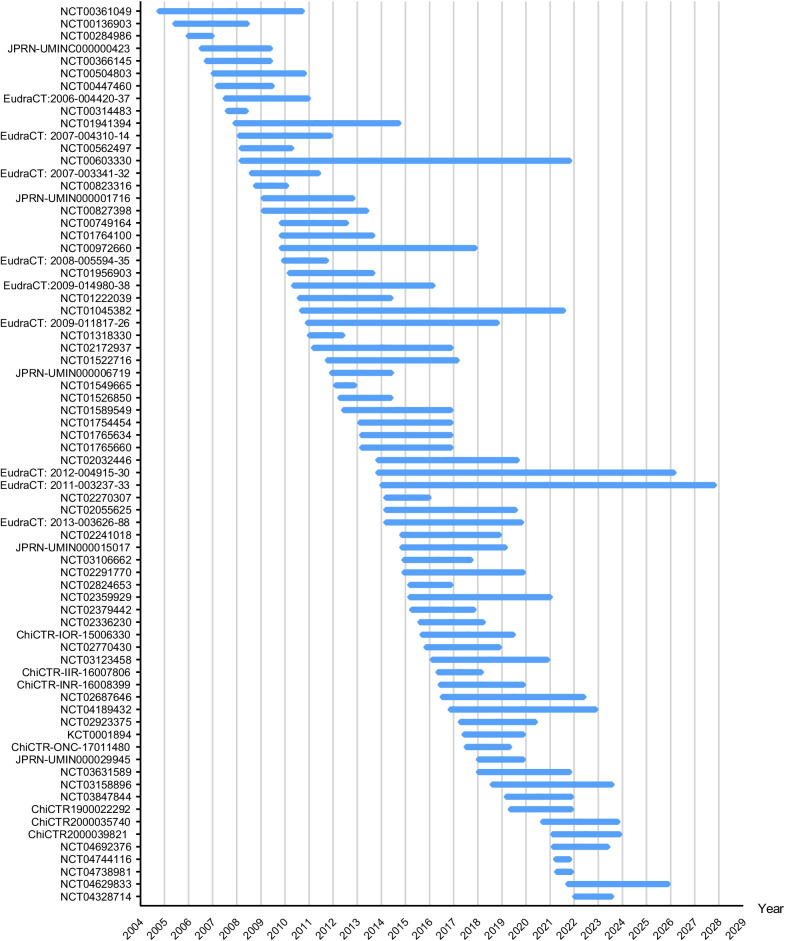
Start and completion time of each clinical trial involving MSC prevention or therapy for GVHD. Because the status of clinical trials No.50 and No.51 is “no longer available” and trial No.52 is “withdrawn”, the start and completion times were not shown in https://clinicaltrials.gov/. Trials No.67, No.68, No.69 and No.70 also did not register the start and completion time

As of November 2021, at least one new clinical trial involving the use of MSCs to mitigate GVHD has been registered every year from 2004 to 2021, with a peak of 8 studies commencing in 2007. As shown in Fig. 3, clinical trials of MSCs to prevent or treat GVHD have gradually increased from 2004 to 2007 and remained steady from 2008 to 2017, but significantly decreased from 2018 to 2020, perhaps due to the increased international scrutiny of clinical trials using MSCs. In 2018, two clinical trials using MSCs led to blindness in patients in the USA, resulting in an overall decrease in the number of clinical trials involving MSCs in the following three years and the number of clinical trials targeting MSCs for GVHD prevention or treatment. Figure 4 lists the start and completion dates for each clinical trial. The duration of clinical trials ranged from 9.5 months to 14 years. The mean duration was 4.19 ± 2.70 years.

**Fig. 5:**
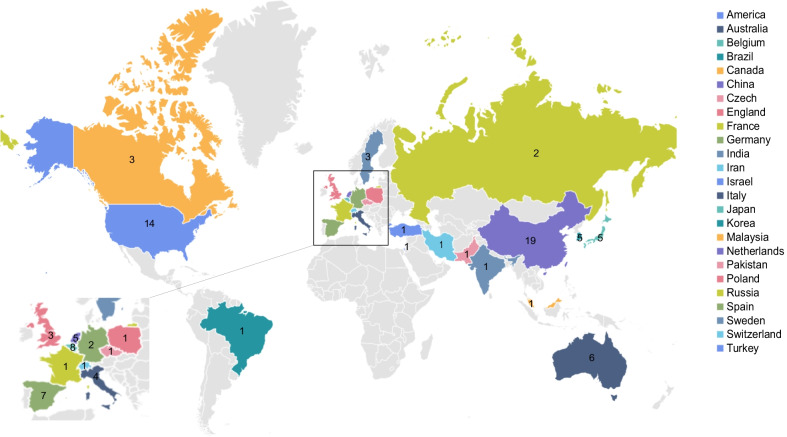
Schematic diagram of location distribution of clinical trials involving MSC prevention or therapy for GVHD

The clinical trials were conducted in 25 countries. China hosted the largest number, with 19 trials conducted, followed by the USA, which hosted 14 trials. Belgium was in third place, having hosted or participated in 8 trials. These clinical trials covered all continents except Africa and Antarctica (Fig. 5). The results are, therefore, of reference value.

**Fig. 6:**
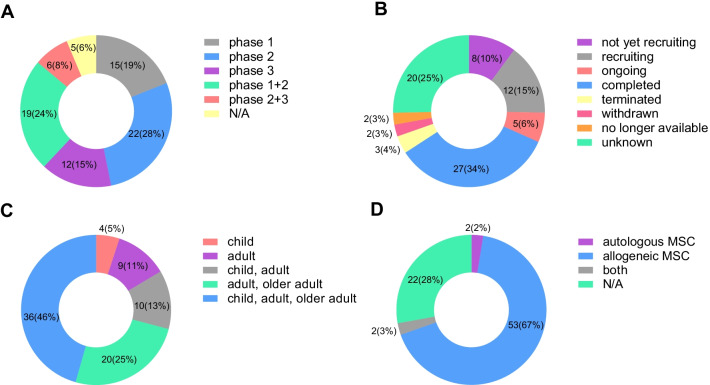
Characteristics of clinical trials involving MSC prevention or therapy for GVHD. **A** Phase of clinical trials; **B** status of clinical trials; **C** subject composition of clinical trials; **D** MSCs origins of clinical trials. *N/A*: not reported

There were 15 phase 1 clinical trials, 22 phase 2 trials, and 19 phase 1 + 2 trials, accounting for 19%, 28%, and 24%, respectively. The three phases of trials accounted for 71% of all included. Therefore, most trials are in the early phases, and the results from these trials need to be further tested in advanced-phase trials. There were 12 phase 3 trials and 6 phase 2 + 3 trials (Fig. 6A).

The status of clinical trials includes recruiting, ongoing, and completed trials. Among the enrolled 79 trials, 12 were still recruiting, 5 were ongoing, and 27 were completed. Notably, three trials were terminated: No.12, due to unavailability of cellular product of bone marrow-derived MSCs (BMMSCs); No.23, due to new requirements for study approval by the Swedish Medical Products Agency; and No.39, due to very slow recruitment. Clinical trials No.29 and No.52 were withdrawn, while trials No.50 and No.51 were no longer available for unknown reasons (Fig. 6B).

Patients were categorized as children under 18 years old, adults between 18 and 60 years old, and adults over 60 years old. Nearly half of the clinical trials had no pre-defined age limit and included patients of all ages. Four clinical trials were designed specifically for children, whereas 20 clinical trials did not enroll children. Ten trials excluded older patients (Fig. 6C).

Although there are reports of MSCs isolated from patients with active acute lymphoblastic leukemia (ALL), acute myeloid leukemia (AML), multiple myeloma (MM), and pre-existing or evolving GVHD [29], it usually takes several weeks for autologous MSCs to be expanded in vitro to produce sufficient numbers of cells for therapeutic use, indicating infeasibility for use in aGVHD patients. In contrast, allogeneic MSCs originate from healthy donors, and physicians can preliminarily predict the immunosuppressive ability of MSCs from the results of in vitro potency assays and select batches suitable for the patient’s condition. Of the 79 clinical trials included in this study, 53 used allogeneic MSCs, 2 used autologous MSCs, and 2 used both. Only 2% of the trials used autologous MSCs in GVHD therapy (Fig. 6D), aligning with the findings of survey on cellular and engineered tissue therapies in Europe [30]. This represents a shift from autologous to allogeneic MSCs in clinical trials, since allogeneic MSCs are instantly available off-the-shelf.

**Fig. 7:**
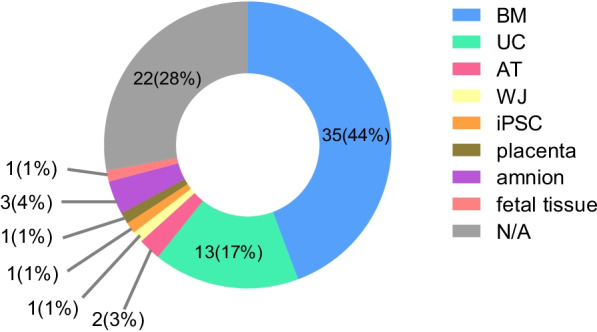
The sources of MSCs used in clinical trials to prevent or treat GVHD. *BM* bone marrow, *UC* umbilical cord, *AT* adipose tissue, *WJ* Wharton’s jelly, *iPSC* induced pluripotent stem cells, *N/A* not reported

Fifty-five clinical trials elucidated the tissue sources of their MSCs. Amongst these, BMMSCs were the most commonly used, followed by umbilical cord-derived MSCs (UCMSCs), amnion-derived MSCs, and adipose tissue-derived MSCs (ATMSCs). Other tissue sources included Wharton’s jelly (WJ), placenta, and fetal tissue (Fig. 7). Notably, clinical trial No.35 used induced pluripotent stem cells (iPSCs)-derived MSCs for the treatment of aGVHD because the indefinite proliferation of pluripotent stem cell lines could overcome the poor scalability of primary MSCs and help avoid inconsistent clinical trial results due to inter-donor variability.

**Fig. 8:**
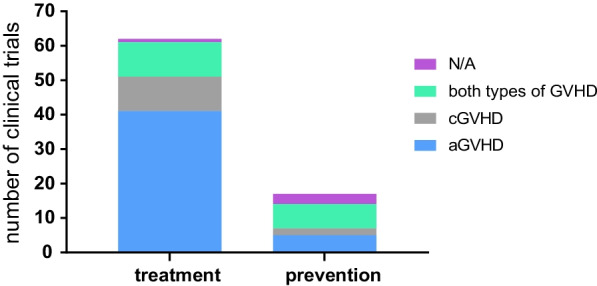
Role of MSCs according to the different subtypes of GVHD in all clinical trials involving MSC prevention or therapy for GVHD. *N/A* not reported

There were 79 clinical trials in which MSCs were used for GVHD; in 17 and 62, MSCs were used to prevent and treat GVHD, respectively. In trials involving using MSCs to treat GVHD, there were 41 cases of aGVHD, accounting for 52% of all the trials, 10 cases of cGVHD and 10 cases of both acute and chronic GVHD. In trials using MSCs to prevent GVHD, there were 5 cases of aGVHD, 2 cases of cGVHD and 7 cases of both (Fig. 8). The safety and efficacy of MSCs in the treatment of aGVHD were the primary focus of these trials.

## Results of clinical trials

Le Blanc et al. [31] reported a case of a 9-year-old boy with SR grade IV aGvHD who received MSCs twice after HSCT, with significant relief of symptoms after each infusion. The boy survived for more than 1 year. In contrast, 24 grade IV aGvHD patients from the same center who did not receive MSCs died an average of 2 months after HSCT. These encouraging results sparked interest in MSC therapy for GVHD, and a multicenter study conducted to determine whether MSCs could ameliorate GVHD yielded satisfactory results [32]. Fifty-five patients with SR aGvHD were treated, 30 of which showed complete response (CR). Moreover, these CR patients had a 55% overall survival rate for 2 years. Since then, more clinical trials using MSCs to prevent or treat GVHD have been conducted, most with promising results. As summarized in Tables 1 and 2, nearly all enrolled patients had grade II–IV aGVHD or moderate to severe cGVHD, and most were SR. Both pediatric and adult patients were included, with ages ranging from 2 months to 72 years. The highest 28-day overall response (OR) rate reached 100% (clinical trials No.44 and No.53), and CR reached 87.5% (trial No.44), much higher than that of GVHD with steroid therapy (OR, ~ 50%; CR, ~ 30%) [33]. It is difficult to compare the overall survival (OS) rate due to differing deadlines of survival observation in different clinical trials, but in nearly all clinical trials, the OS of patients who responded to MSCs was significantly higher than that of those who did not.

**Table 1:** Results of clinical trials using MSCs to treat GVHD

Clinical trial	Median age (range)	Condition	MSC administration protocol		28d response rate (%)	Other time points response rate (%)	Survival rate (%)	Follow-up time	Reference
			MSC frequency	MSC dosage (× 10^6^/kg)					
No.1	27.8y (1–65)	SR grade II–IV GVHD	3 (children: 2–7 adults: 2–11)	1.5 (0.8–3.1)	OR:67.5 CR:27.5	N/A	1y:50 2y:38.6 median survival time:1.1y	2.8y	Introna et al. [54]
No.2	44.9y (1.3–68.9)	SR grade II–IV aGVHD	3 (1–4)	1.8 (0.9–2.5)	CR:25	N/A	1y:44	150d	Te Boome et al. [71]
No.7	21–66y	refractory acute or chronic GVHD	aGVHD:1–4 cGVHD:1–3	0.2–2.9	N/A	exact time unknown:aGVHD:OR:70 CR:10 cGVHD:OR:50 CR:12.5	N/A	N/A	Pérez-Simon et al. [55]
No.12	≤ 18y	N/A	N/A	N/A	N/A	N/A	N/A	N/A	https://clinicaltrials.gov/ct2/show/results/NCT02379442?term=02379442&draw=2&rank=1
No.14	58y (5–69)	grade II–IV SR aGVHD	1/2	1–2 or 3–4	OR:40.6 CR:21.9 (30 d)	90d:OR:46.9 CR:31.2	90d:30.3 1y:18.2	1y	Servais et al. [96]
No.23	50y (20–61)	moderate to severe refactory cGVHD	6–9	2	N/A	time points varies from different patients:OR:54.5	N/A	76m	Boberg et al. [75]
No.27	7y (0–17)	grade II–IV SR aGVHD(excluding skin-only grade II)	8	2	OR:70.4 CR:29.6	56d:OR:59.3 CR:31.5 100d:OR:70.4 CR:44.4	100d:74.1 180d:68.5	180d	Kurtzberg et al. [37]
No.32	group A:51y (24–60) group B:46y (36–60)	moderate or severe cGVHD	1	group A:1 group B:3	N/A	20w:OR:71.4 CR:35.7 42w:OR:71.4 CR:42.9 56w:OR:71.4 CR:57.1	56w:71.4 median survival time:45.3w	56w	Jurado et al. [102]
No.35	18–70y	grade II–IV SR aGVHD	2	cohort A:1 cohort B:2	cohort A:OR:62.5 CR:12.5 cohort B:OR:85.7 CR:57.1 total:OR:77.3 CR:33.3	100d:cohort A:OR:87.5 CR:50 cohort B:OR:85.7 CR:57.1 total:OR:86.7 CR:53.3	100d:cohort A:87.5 cohort B:85.7 total:86.7	100d	Bloor et al. [39]
No.42	6 m–70y	SR grade II–IV aGVHD	8 (+ 4)	2	OR:remestemcel-L group:58 placebo group:54 *p* = 0.59	N/A	180d:remestemcel-L group:34 placebo group:42	180d	Kebriaei et al. [64]
No.42	N/A	SR aGVHD	8 (+ 4)	2	N/A	100d:OR:remestemcel-L group:82 placebo:73 *p* = 0.12	N/A	N/A	Martin et al. [88]
No.43	57.5y (35–73)	de novo high risk or SR grade I–IV aGVHD	2	low dose group:2 high dose group:10	OR:70 CR:40	N/A	100d:90 180d:60	N/A	Soder et al. [103]
No.44	52y (34–67)	grade II–IV aGVHD	2	low dose group:2 high dose group:8	low dose group:OR:87.5 CR:87.5 high dose group:OR:100 CR:66.7 total:OR:94 CR:77	N/A	90d:71.0	90d	Kebriaei et al. [87]
No.45	36y (21–52)	refractory acute and chronic GVHD	1	1.0	N/A	1w:OR:40	5w:90.9	5w	Yi et al. [58]
No.50	9.6y (0.3–18.2)	grade II–IV SR aGVHD	11 (1–24)	2	OR:65.1 CR:14.1	100d:OR:51.5 CR:32.8	100d:66.9	100d	Kurtzberg et al. [37]
No.50	7.8y (0.2–17.5)	grade II–IV SR aGVHD	10 (1–20)	2	OR:61.3	100d:OR:77	100d:57.3	100d	Kurtzberg et al. [67]
No.53	group1:54.5y (0.9–65.6) group2:48.9y (1.6–72.4)	grade II–IV aGVHD	group1:1 (1–5) group2:2 (1–6) *p* = 0.002	group1:2.0 (0.9–2.8) group2:1.2 (0.9–2.9) *p* < 0.001	group1:OR:58.8 CR:29.4 group2:OR:100 CR:52.4 *p* = 0.013 group1 SR patients:OR:46 group2 SR patients:OR:100	N/A	1y:group1:47 group2:76 *p* = 0.016 group1 SR patients:31 group2 SR patients:73 *p* = 0.02	N/A	Ringden et al. [56]
No.53	49y (1.6–72)	grade II–IV aGVHD	2 (1–6)	1.2 (0.9–2.9)	OR:100 CR:52.4	56d:OR:95	1y:81 2y:67 4y:57 SR patients:4y:55	4y	Sadeghi et al. [104]
No.64	52y (4–62)	SR grade II–III aGVHD	8 (5–12)	2	OR:93 CR:57	24w:OR:93 CR:85.7	24w:78.6 96w:57.1	96w	Muroi et al. [34]
No.65	33y (5–66)	SR grade III–IV aGVHD	4–12	2	OR:60 CR:24	12w:OR:60 CR:52 24w:OR:60 CR:48 36w:OR:52 CR:44	52w:48	52w	Muroi et al. [35]
No.66	41.5y (17–68)	severe SR cGVHD	4	1	N/A	8w:PR:100	exact time unknown:90	N/A	Kim et al. [28]
No.69	aGVHD:21–61y cGVHD:31–53y	grade II–IV SR aGVHD;SR cGVHD	aGVHD:2–19 cGVHD:2–11	1.7–2.3	N/A	exact time unknown:aGVHD:OR:92 CR:58 cGVHD:OR:57 CR:29	aGVHD:3y:55 cGVHD:median survival time:8m	N/A	Herrmann et al [74]

**Table 2:** Results of clinical trials using MSCs to prevent GVHD

Clinical trial	Median age(range)	MSC frequency	MSC dosage	aGVHD morbidity (%)	cGVHD morbidity (%)	Survival rate (%)	Follow-up time	References
No.21	Standard prophylaxis group:30y (18–60) Standard prophylaxis + MSCs group:37y (20–63)	1	1 × 10^6^/kg	100d grade II–IV aGVHD:Standard prophylaxis group:29.4 Standard prophylaxis + MSCs group:9.4 *p* = 0.041	N/A	exact time unknown:Standard prophylaxis group:61.8 Standard prophylaxis + MSCs group:81.2 *p* < 0.05	N/A	Kuzmina et al. [42]
No.21	34y (17–63)	1	1.2 (0.9–1.65) × 10^6^/kg	100d grade II–IV aGVHD:Standard prophylaxis group:20.5 Standard prophylaxis + MSCs group:10.2	N/A	N/A	N/A	Shipounova et al. [43]
No.34	MSC group:58y (21–69) historic group:55y (10–69)	1	N/A	100d grade II–IV aGVHD:MSC group:35 historic group:56 grade IV aGVHD:MSC group:10 historic group:19	1y moderate/severe cGVHD:MSC group:65	1y:MSC group:80 historic group:44 *p* = 0.02	560d	Baron et al. [93]
No.38	MSC group:6.9y (3.3–12.5) historic group:9.5y (8.4–18.7)	1	1/5 × 10^6^/kg	grade I–II aGVHD:MSC group:57.1 historic group:62.5 *p* = 0.83 grade III–IV aGVHD:MSC group:14.3 historic group:0 *p* = 0.36	extensive cGVHD:MSC group:14.3 historic group:50.0 *p* = 0.27	2y:MSC group:85.7 historic group:55.6 *p* = 0.15	N/A	Lee et al. [94]
No.60	N/A	3.7 (2–4)	3 × 10^7^/100 mL/m	N/A	2y:MSC group:27.4 control group:49.0 *p* = 0.021	2y:MSC group:66.1 historic group:61.3 *p* > 0.05	51m	Gao et al. [92]

d represents days; w represents weeks; y represents years

**Fig. 9:**
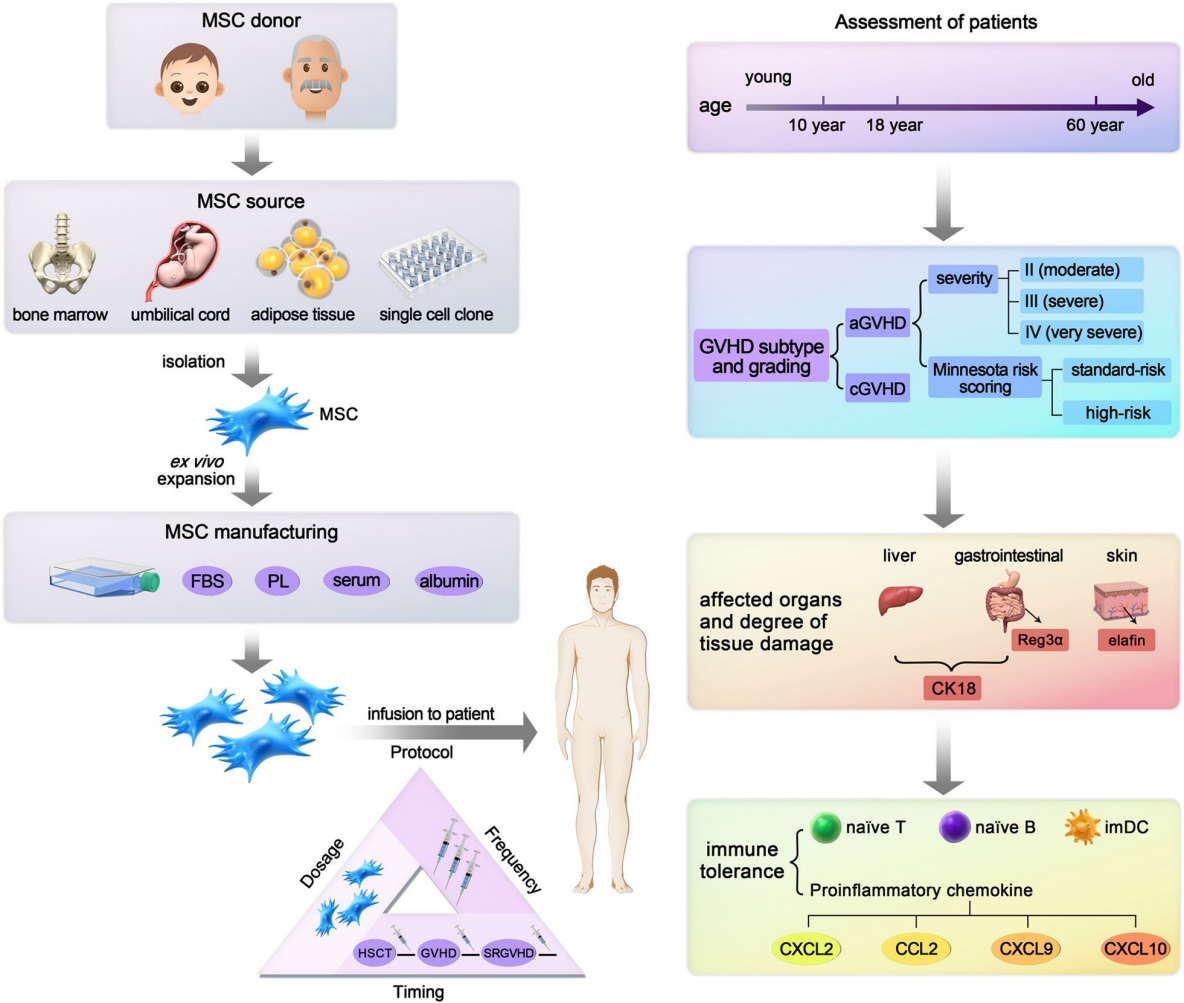
Reasons for the inconsistent results of clinical trials involving MSC prevention or therapy for GVHD. In terms of MSCs, it includes the donor age, tissue source and manufacturing process, mainly the composition of medium; in terms of patients, including age, subtype and grading of GVHD, tissue damage degree of affected organs, and immune tolerance status of patients; in clinical trial design, including the timing, dosage and frequency of MSCs

In contrast to these desirable outcomes, low response rates or no benefit over placebo has been documented in clinical trials, among which, the CR of trial No.7 was only 10%, the OR of trial No.14 was only 40% and 1-year OS only 18%. Moreover, trials yielding negative results are often not published. The large-scale randomized trial (No.42) failed to demonstrate that Prochymal™ (Ex vivo Cultured Human Mesenchymal Stem Cells) was superior to placebo in all groups; hence, it was not approved in the USA. Subsequent subgroup analyses, however, confirmed that MSCs were more effective than placebo in pediatric patients. In the following year, clinical trial No.50 using Remestemcel-L (Prochymal®, Osiris Therapeutics Inc., Columbia, MD) to treat pediatric GVHD was conducted in Canada, New Zealand, and other countries, achieved satisfactory results, leading to the approval of Prochymal in Canada for pediatric GVHD on May 17, 2012. Later in Japan, phase I/II and II/III clinical trials No.64 and No.65 applying this product to treat SR aGVHD achieved satisfactory results [34, 35]. The Japanese Ministry of Labor and Welfare approved its marketing on September 15, 2015. However, in the USA and Europe, patients with aGVHD can obtain MSC treatment only by participating in clinical trials [36]. In 2015, clinical trial No.27 was conducted in the USA to validate the effectiveness of Remestemcel-L in pediatric aGVHD patients. The 28-day OR reached 70.4% and the 180-day OS reached 68.5% [37]. With publication of these favorable results, Mesoblast, Inc. applied to FDA for the listing of Remestemcel-L for SR aGVHD treatment in children [38]. Meanwhile, clinical trial No.44 to confirm the effectiveness of Prochymal in adult patients is ongoing. The complex biological variability of MSCs, combined with the complex pathogenesis of GVHD, diverse patient characteristics, and the diversity of administration protocols, contributed to the discrepancies between clinical trial outcomes (Fig. 9).

## Reasons for erratic results of clinical trials

### Heterogeneity of MSCs

#### MSC donors

MSC gene expression, proliferation, differentiation, and colony-forming capacity vary markedly among donors [39]. The same research team conducted a phase II study (No.2) and a hospital exemption program. As the two studies shared the same protocol, researchers pooled the two cohorts together, covering a total of 102 patients suffering from SR aGVHD receiving MSC infusions derived from 12 different donors. Researchers observed a significant survival benefit for patients treated with MSCs derived from young (< 10 years old) compared to older (> 10 years old) donors. In the multivariate analysis, MSC donor age remained a significant predictive independent variable for OS (*p* = 0.025) [40]. Researchers performed a transcriptome analysis of MSCs derived from young donors compared with older donors and found 104 differentially expressed genes (DEG). Among them, 31 genes were up-regulated and 73 were down-regulated, suggesting that MSCs from younger donors have unique molecular characteristics [41]. Further analysis of these DEGs showed that Eph receptor B2 (EphB2), involved in the inhibition of T cell activity, SRY-box transcription factor 11 (SOX11), and tripartite motif containing 59 (TRIM59), which promotes MSC differentiation, were associated with the outcomes of GVHD treated by MSCs.

Kuzmina and Shipounova conducted a phase II prospective randomized clinical trial (No.21), to determine whether MSCs could reduce the incidence of GVHD after HSCT based on standard prophylaxis. Results showed that after the administration of MSCs, the incidence of grade II–IV aGVHD 100-days after transplantation was significantly lower than that of the standard prophylaxis group (*p* = 0.041), while the OS was significantly higher than that of the standard prophylaxis group (*p* < 0.05). This data indicates that MSCs can effectively prevent the incidence of aGVHD after HSCT [42]. However, some patients in the standard prophylaxis + MSCs group developed aGVHD, and researchers compared these MSCs with those that could effectively prevent GVHD and found differences in the relative expression levels of some genes. The expression level of pro-mitotic fibroblast growth factor receptor 1 (FGFR1) was lower in MSCs which could not prevent GVHD, and the expression levels of peroxisome proliferator-activated receptor gamma (PPARG) and insulin like growth factor 1 (IGF1), biomarkers associated with MSC differentiation and senescence, were significantly higher, indicating that senescent MSCs are worse at preventing aGVHD [43]. Thus, the age of the MSC donor can directly affect the efficacy of MSCs in GVHD prophylaxis and therapy, and unclear donor profiles could result in inconsistent clinical trial results.

#### MSC source

MSCs are mainly divided into BMMSCs, UCMSCs, ATMSCs, Wharton’s jelly MSCs, placenta MSCs, and fetal liver MSCs (FLMSCs) [44, 45]. MSCs from different sources share common characteristics, but also display phenotypic and functional differences [46]. The proportion of BMMSCs is the highest in clinical trials applying MSCs to prevent or treat GVHD, accounting for 43% (Fig. 7). However, the process of obtaining BM is invasive and poses risks to the donor, such as bleeding and infection at the puncture sites. UCMSCs can be obtained more easily, and express embryonic stem cell markers such as Tra-1–60, Tra-1–81, and stage-specific embryonic antigen-1 and 4 [47]. They proliferate faster in vitro [48] and show reduced immunogenicity and stronger immunosuppressive effects compared to BMMSCs [49], making them ideal candidates for GVHD. Likewise, the broad accessibility and the simple procedure for obtaining ATMSCs make them an attractive option for cell therapy [50]. Moreover, in the context of HSCT, ATMSCs may be more effective than BMMSCs because although both have similar immunosuppressive effects on T cell proliferation, ATMSCs preserve NK cell activity and promote graft versus leukemia (GVL) effect [51].

So far, no clinical trials have directly compared the efficacy of MSCs from different sources; the most appropriate source for GVHD remains unclear. A preclinical study compared the ability of BMMSCs, ATMSCs, and UCMSCs to treat GVHD in NSG-HLA-A2 mice infused with peripheral blood mononuclear cells from non-HLA-A2 donors. This model mimics GVHD pathogenesis in humans [52]. The results showed that UCMSCs induce Tregs more potently than BMMSCs, while BMMSCs induce higher levels of IL10^+^CD4^+^, IFNγ^+^CD8^+^ cells and Th17 cells. Moreover, researchers observed a higher expression of HLA-DR in Tconv cells in the BMMSCs group than in the UCMSCs group on day 28. Therefore, unlike UCMSCs and ATMSCs, BMMSCs in an inflammatory environment potentially induce a rapid proinflammatory reaction before exerting their immunosuppressive properties. Thus, the BMMSCs, while the most widely used MSCs in clinical trials, may not be optimum.

Yu et al. compared the immunomodulatory properties of FLMSCs and BMMSCs and demonstrated that FLMSCs played a more potent and lasting immunosuppressive function than BMMSCs by inhibiting the proliferation and activation of CD4^+^ and CD8^+^ T cells and inducing T regs [45]. However, clinical trials of FLMSCs for GVHD prophylaxis or therapy have not been conducted. Currently, there has been one clinical trial (No.36) evaluating the safety and efficacy of fetal MSCs in the treatment of GVHD, but which fetal tissue was used in this trial has not been specified, and the results have not been reported yet. Based on the strong and persistent immunosuppressive ability of FLMSCs, clinical trials on the prevention or treatment of GVHD using FLMSCs are expected to be carried out as soon as possible.

In addition, researchers should continue to compare the immunosuppressive function of MSCs from different sources, clarify the mechanisms of their differences, and then to verify the findings in clinical trials, and finally determine the most suitable MSC source for GVHD.

#### Variability in MSC manufacturing

The quality of MSCs is influenced by many factors during isolation and cultivation, among which expansion conditions, mainly different medium compositions, are crucial. Researchers compared the most commonly used media supplements, fetal bovine serum (FBS), and human-derived blood products, including human serum and platelet lysate (PL), and their effects on the immunomodulatory potency of MSCs [47]. The use of PL in MSC expansion has been shown to maintain its immunomodulatory potential [53]. In clinical trials using MSCs expanded in human serum and/or PL to treat GVHD, including No.1, No.7 and No.53, the overall response (OR) rate measured on day 28 ranged between 58.8 and 71.4% [54–56]. However, a meta-analysis showed that SR aGVHD patients achieved slightly better OR when FBS was used for MSC culture than human serum [57], because human serum contains functional complements, which bind to MSCs and cause their lysis [56]. Therefore, researchers searched for more suitable supplements for MSC resuscitation and culture. In clinical trial No.53, researchers replaced serum with albumin and found that, compared with the addition of serum, the cell viability of albumin group (serum group: 90%; albumin group: 95%; *p* < 0.001) and the patient's 28-day OR (serum group: 58.8%; albumin group: 100%; *p* = 0.013) and 1-year OS (serum group: 47%; albumin group: 76%; *p* = 0.016) were significantly higher. However, the MSCs used in this trial came from placenta, and there was no comparison between albumin and FBS. Therefore, in future clinical trials, this method should be first applied to BMMSCs or UCMSCs, more commonly used in trials, and compare the response rate and survival rate of GVHD patients to MSCs supplemented with albumin and FBS directly.

The more homogeneous the MSC product, the better the efficacy, the less treatment dose required, and therefore the less likely it is to cause adverse events. However, MSCs used in current clinical trials are mostly non-clonal; such MSCs may contain other cell types in the final product. There are some concerns regarding the heterogeneity caused by these non-clonal MSCs. With advancements in cell isolation and culture technology, researchers can now generate single-cell-derived clonal MSC lines. Clinical trial No.45, which used bone marrow-derived clonal MSCs to treat GVHD, was completed. The OR was 40% at 1 week after treatment with only one dose of MSCs at 1 × 10^6^ MSCs/kg. During the 5-week post-infusion, no serious adverse events occurred and the OS reached 91% [58]. Since iPSC-derived MSCs (CYP-001) can effectively treat the aGVHD model of humanized mice [59], researchers have conducted a phase 1 multicenter clinical trial (No.35) to treat SR aGVHD patients with CYP-001, which was the first clinical trial to be completed using iPSC-derived MSCs. This has significant implications for GVHD and other diseases that can be treated with primary MSCs. The results showed that the 28-day OR reached 73.3%, and the 100-day OS reached 86.7%, which was no less than BMMSCs [39]. Other clinical trials (No.36 and No.48) to evaluate the safety and efficacy of clonal MSCs in the therapy of GVHD are underway. Even small variations in the isolation and culture protocol can significantly influence the yield, quality, and composition of the MSC population, leading to inconsistent results in clinical trials. A better understanding of the impact of preparation process on the immunomodulatory properties of clinical-grade MSCs will greatly promote future therapeutic applications.

### Various patient characteristics

The role of MSC recipients in predicting clinical outcomes is crucial [60]. When MSCs from the same donor were used to treat several patients, the response level of patients was different, suggesting a key role of patient characteristics in determining the response to MSC treatment [61].

#### Age of patients

The age profile of patients profoundly influences the outcome of clinical trials [62, 63]. A multicenter randomized clinical trial (No.42) was conducted to evaluate the effectiveness of Prochymal (Ex vivo Cultured Human MSCs) in addition to second-line therapy in the treatment of SR aGVHD during 2006–2009 [64]. A total of 260 patients, ranging from 6 months to 70 years old, were enrolled and received Prochymal or placebo. The durable complete response (DCR) in the Prochymal group was not greater than that in the placebo group (35% versus 30%; *p* = 0.42). The investigators divided the patients into subgroups, analyzed the outcomes further, and found that pediatric patients had a higher OR with Prochymal compared to placebo (64% versus 23%; *p* = 0.05). Upon comparing the age composition of Prochymal group and placebo group, we found that children were underrepresented in the former. Patients younger than 18 years old accounted for 8.6% in Prochymal group and 16.0% in placebo group. This imbalance between treatment arms probably resulted in failure of MSCs to show better efficacy than the placebo. Six years after the completion of trial No.42, trial No.27 of the same ex vivo expansion MSCs (remememcel-L) for the treatment of SR aGVHD specifically in children was conducted, which enrolled only patients aged 2 months to 17 years, showing an OR of 70.4% at 28 days, higher than 45% of prespecified control (*p* = 0.0003), and the OS at 180 days was 68.5%. Therefore, as a choice for children with SR aGVHD, remememcel-L has a significant effect. Compared with trial No.42, the doses and frequency of MSCs were identical in both clinical trials, except for the age composition of the patients. In both children and adults, MSC therapy reduced the proportion of pro-inflammatory Th1 cells, but could only increase the level of Treg in children [65], not in adults [66]. This makes the Th1/Treg ratio of pediatric patients further develop in the direction of anti-inflammatory, explaining why children have a higher OR to MSCs than adults. In addition, children have a higher response rate than adults probably because children are more likely to receive MSCs as a second-line therapy directly after SR. In contrast, adults are more often switched to other immunosuppressants, and are not usually administered with MSCs until they experience two to six lines of failure [54]. Patients enrolled in trial No.27 did not receive any other immunosuppressive agents after SR, whereas all patients in No.42 received other second-line therapies. Certainly, trial No.27 still has some shortcomings in study design, such as open-label, unknown randomization, no placebo control, and only 54 included patients. Besides trial No.27, clinical trials specifically for children registered on http://clinicaltrials.gov were No.12 and No.50. Although the latter achieved satisfactory results, with the 28-day OR reaching 65.1% and 100-day OS reaching 66.9% under the background that 190 (78.8%) patients received ≥ 3 second-line therapies before initiation of MSC therapy, and demonstrated that OR of patients younger than 10 years old was significantly higher than that of pediatric patients aged 10 years or more (71.2% vs 58.6%; *p* = 0.041) [67], these two clinical trials were terminated and are no longer available. Only clinical trial No.54 registered on ICTRP was specifically for children and it is currently recruiting subjects. Therefore, the next step is to conduct large-scale, randomized, blinded, placebo-controlled clinical trials to verify the efficacy of MSCs in preventing or treating SR aGVHD in pediatric patients.

#### Biomarkers of patients

Dander et al. [65] identified and validated interleukin 2 receptor alpha (IL-2Rα), tumor necrosis factor receptor (TNFR) I, and elafin as reliable to predict the response of patients with GVHD to MSCs. After MSC infusion, the patients with CR showed a significantly different variation trend from those with partial response (PR) or no response (NR). Although some patients had similar GVHD clinical scores, they had different pre-MSC treatment biomarker levels. Patients who did not respond to MSCs had higher levels of TNFRI, IL-2Rα, elafin, cytokeratin fragment 18 (CK18), and suppression of tumorigenicity 2 (ST2) than those who responded prior to MSC infusion, demonstrating that these markers may be reliable indicators for predicting the response to MSCs [68]. There are even markers that can predict the response of the sole GVHD-affected organ to MSCs. For example, elafin is specific to the skin, regenerating islet-derived 3α (Reg3α) is specific to the gastrointestinal system [69], and CK18 is a tissue damage marker for liver and intestine [70]. Clinical trial No.1 assessing the feasibility of MSCs in SR GVHD patients, demonstrated the predictive value of IL-2Rα. When comparing responders versus non-responders, a significant difference was found in IL-2Rα plasma levels, suggesting the valuable role of IL-2Rα for MSC response evaluation [54]. However, the above markers can only reflect the degree of tissue damage caused by GVHD, and are not directly related to the role of MSCs in the therapy of GVHD. The phase II prospective clinical trial (No.2) evaluating the efficacy of MSCs in the treatment of SR aGVHD and the predictive value of these biomarkers for the prognosis of MSC therapy, did not show that the above markers are associated with 28-day CR [71], and therefore more predictive markers need to be developed.

Another approach is to monitor cellular pathways that have been reported to be mediators of MSC immunosuppression. Hinden et al. [72] reported that the number of lymphocytes, especially T cells and NK cells, was significantly higher in responders than in non-responders before MSC administration. The more inflammatory milieu of responders is conducive to MSC “licensing” and thus is beneficial for MSCs to play an immunomodulatory role [73]. However, clinical trial No.23 detected the lymphocyte subsets of cGVHD patients before MSC treatment and found no difference in the number of lymphocytes or T cells between responders and non-responders, but found that the number and proportion of naïve T and B cells were significantly higher in responders than in non-responders. Moreover, these naive lymphocytes were mobilized by thymus rather than expanded by peripheral lymphocyte pool, suggesting that patients with better thymus function (higher proportion of CD31^+^ cells among naïve CD4^+^ T cells) were more likely to respond to MSCs. Investigators of this trial also tested the levels of chemokines in cGVHD patients before and after MSC infusion and found that the levels of pro-inflammatory chemokines CXCL9, CXCL10, and CXCL2 in non-responders continued to increase from pre- to 5 months after treatment, while those in responders remained stable, with a further reduction in the levels of CXCL2 and CCL2. These changes may not be MSC-specific, but suggest that the systemic inflammatory environment is declining in respondents, while progressing in non-responders. Clinical trial No.2 reported that the proportion of immature dendritic cells (DCs) was significantly higher in responders than in non-responders following MSC infusion; the decreased ability of immature DCs to secrete TNF-α suggests a state of immune tolerance in MSC-responders [71].

The above findings indicate that patients play an important role in determining the prognosis of MSC therapy. Detecting markers to elucidate the ongoing inflammatory status can identify patients more likely to respond to MSCs and predict treatment outcomes early. Future research should explore the mechanism by which MSCs exert immunosuppressive effects in the context of GVHD, search for key markers, and verify the predictive value of these markers in large-scale prospective clinical trials. In addition, we will continue to explore other patient-derived reasons that may induce the inconsistency of MSC therapy for GVHD.

### Heterogeneity of disease

#### Subcategory of GVHD

Herrmann et al. [74] performed a phase I clinical trial (No.69) using BMMSCs to treat patients who developed SR GVHD. Among twelve patients with aGVHD, seven achieved CR, four PR, and one had no response. Among seven patients with cGVHD, two achieved CR, two PR, and three gave no response. Five deaths occurred in this group, and the median survival for cGVHD was only eight months. In contrast, in the acute group, the three-year survival rate was 55%, indicating that the median survival for aGVHD was longer than three years, much longer than that of the chronic group.

However, MSCs are not completely useless against cGVHD. Clinical trial No.32, evaluating the effect of MSCs in the treatment of moderate and severe cGVHD, performed in Spain in 2010, achieved striking results. The 20-week OR was 71.4%, higher than the 28.6% in the historical control group. The 20-week CR was 35.7%, gradually increased over time, and reached 57.1% at 56 weeks. Moreover, the OS was 71.4%, indicating that MSCs have a better therapeutic effect on cGVHD. The following year, clinical trial No.23, performed in Sweden, also evaluated the efficacy of MSCs in the treatment of cGVHD [75]. The results were encouraging, with an OR of 54.5% at the end of treatment with variable number of MSC infusions, ranging from 20 to 48w. The median follow-up time of this trial was 76 months. In the last follow-up, 1/3 of all patients who obtained OR had discontinued all immunosuppressants, 1/3 no longer required steroids, and calcineurin inhibitors were also being reduced, suggesting that MSC therapy for cGVHD can induce a sustained response. Clinical trial No.60 evaluated the effect of MSCs on the prevention of cGVHD. The 2-year-cumulative incidence of cGVHD in MSC group was 27.4%, lower than the 49% in historical control group (*p* = 0.021). The analysis of lymphocyte subsets in the two groups showed that after MSC infusion, production of Treg was induced, increasing the number and proportion of memory CD27^+^ B lymphocytes and reducing the number of NK cells, thereby reducing the incidence of cGVHD. The lung was the main organ affected in cGVHD; lung damage was irreversible and often did not respond to treatment, with fibroproliferative changes associated with increased Th2 cells [76]. The proportion of Th2 cells decreased after prophylactic infusion of MSCs, and no patient developed pulmonary GVHD in MSC group, compared with seven patients in the control group (P = 0.047), indicating that MSCs have a preventive effect on cGVHD. To elucidate these seemingly paradoxical results, researchers need to profoundly understand the pathogenesis of acute and chronic GVHD and the immunomodulatory mechanisms of MSCs in various pathological background.

#### Severity of GVHD

A phase I multicenter clinical trial (No.1) aimed to assess the feasibility of MSCs in 40 SR grade II–IV aGvHD patients showed grade II aGVHD had a statistically better chance of achieving CR compared to higher grades (grades III and IV, with 61.5% and 11.1%, respectively; *p* = 0.002). Moreover, better survival was observed in patients with grade II disease (*p* = 0.0482). No.64 and No.65 were two clinical trials carried out in Japan to determine the efficacy of MSC preparation JR-031 in the treatment of SR aGVHD. The time, frequency, and dose of MSCs administered were consistent in both trials; the former included patients with grade II–III, whereas the latter included patients with grade III–IV. The results showed that the 28-day OR and CR of grade II–III aGVHD patients were 93% and 57%, while the 28-day OR and CR of grade III–IV aGVHD patients were only 60% and 24%, which fully indicated that MSCs had better efficacy in patients with milder aGVHD [34, 35]. Dalowski et al. [77] reported the use of MSCs in salvage therapy in 58 patients with SR aGVHD, of which only 5 showed CR, with a 2-year OS of 17%, which were not superior to those of a historical cohort of patients receiving an alternative salvage therapy. Analysis of failure of the trial pointed to the high proportion of critically ill patients. Most MSC-treated patients (*n* = 46, 79%) presented with grade IV aGVHD, whereas the initial aGVHD grade of patients in the historical control group was less severe, with only 49% presenting with grade IV. There was a significant difference in the severity of aGVHD between the two groups (*p* < 0.004). The imbalance between the treatment arms in GVHD severity at baseline may induce poor outcomes. This means that once the disease causes severe tissue damage, it is difficult to reverse by MSC therapy.

However, the phase III multicenter clinical trial (No.27) investigating the efficacy of MSCs in the treatment of pediatric patients with SR aGVHD, obtained opposite results [37]. The 28-day OR of grade II GVHD patients was 50%, lower than that of grade III and IV (69.6% and 76.0%, respectively). This is because higher levels of inflammatory cytokines elicit stronger anti-inflammatory and immunosuppressive effects of MSCs. Inflammatory cytokines stimulated the differentiation of MSCs to an immunomodulated phenotype. Comparative proteomics analysis of MSCs stimulated with IFN-γ revealed that the expression levels of indoleamine 2,3-dioxygenase (IDO), programmed cell death 1 ligand 1 (PD-L1), intercellular adhesion molecule 1 (ICAM-1), and vascular cell adhesion molecule 1 (VCAM-1) were significantly up-regulated [78]; the whole transcriptome analysis of interleukin-1β (IL-1β) stimulated MSCs indicated that the genes that regulate immune response were significantly up-regulated [79]. In mice with aGVHD and xenogenetic GVHD models, MSCs stimulated by inflammatory cytokines showed better efficacy than unstimulated MSCs, with further reduced GVHD scores and improved survival rate [28, 80]. However, only 6 grade II GVHD patients were included in this trial, and patients with skin-only involvement were excluded. The small sample size and skin involvement, which is an indicator of good prognosis compared with other visceral involvement, might have had an impact on the results. In addition to grading GVHD based on clinical manifestations, Minnesota risk scoring has also been used for judging the severity of GVHD [81]. In clinical trial No.42, a trial comparing the efficacy of Prochymal™ and placebo in the treatment of GVHD, risk stratification was performed for all patients according to Minnesota risk scoring. Among high-risk aGVHD patients, the OR was significantly higher in MSC group than in placebo group (58% versus 37%; *p* = 0.03). However, in standard-risk aGVHD patients, no significant differences were observed between the two groups, indicating that high-risk aGVHD patients responded better to MSCs.

Therefore, large-scale clinical trials, which include stratified comparisons of OR and OS according to different severity of GVHD, are warranted to obtain exact results and to explore the mechanism of MSCs in GVHD with various severity.

#### Organs affected by GVHD

Higher OR and OS are generally observed in cutaneous GVHD than in visceral, namely gastrointestinal or hepatic [8, 67, 82–84]. Ball et al. [85] performed a retrospective analysis of 37 children with SR grade III–IV aGVHD treated with MSCs. In the 30 children with skin involvement, the OR reached 80%, with a CR of 57% and median time to skin resolution of 6 days. Thirty-two children with gastrointestinal involvement took an average of 11 days after the first MSC infusion to a relief of gastrointestinal symptoms. Hepatic involvement was diagnosed in 25 children, and OR was achieved in 18, among whom, reduction in bilirubin levels was evident after a median period of 14 days, and persistent aminotransferase abnormalities were evident in 5 patients. Thus, it was evident that patients with skin involvement responded both better and more rapidly to MSC infusion, while a longer time was needed to observe complete normalization of liver function and gastrointestinal symptoms. However, the gut and liver have been reported to be more responsive to MSCs than skin in one review, but the authors did not cite a clinical trial as an example [36]. Based on this, clinical trial No.27 was conducted, which excluded patients with grade II aGVHD and with involvement limited to the skin, which are considered to be two causes of low response rate, and satisfactory results were achieved [37].

Gut is the organ most frequently involved in GVHD, either alone or simultaneously with skin and/or liver [67, 71, 86]. MSC treatment has a favorable clinical effect on gut aGVHD. Fourteen patients participated in the multicenter, phase I/II clinical trial (No.64) using MSCs for the treatment of SR aGVHD. Of these patients, ten had gut involvement, and of those, all but one showed CR after MSC treatment [34]. Other clinical trials, including No.42, No.44, and No.65 also have yielded satisfactory results in the treatment of gastrointestinal GVHD with MSCs [35, 86–88]. This may be due to the rapid healing effect of MSCs on damaged intestinal epithelium. MSCs are the source of female intestinal epithelial cells [31]. In baboons, intravenously injected MSCs, whether autologous or allogeneic, are distributed in gastrointestinal tissues [89]. Remarkably, although the pathological signs of aGVHD completely disappeared, the symptoms continued to appear in a considerable number of patients with gut aGVHD. These symptoms were caused by villous atrophy after inflammation. This phenomenon suggests that when symptoms persist in patients with gut aGVHD, biopsy is recommended to identify whether the cause is malabsorption after inflammation or active aGVHD [85].

Galleu et al. [62] conducted a retrospective analysis of a cohort of 60 SR aGVHD patients treated with MSCs. Liver involvement was present in 18 patients and absent in 42 patients. Among patients with liver involvement, only 22% (*n* = 4) patients responded. The response rate was 67% (*n* = 28) among those without liver involvement. The authors also performed multivariate regression analysis of factors affecting the response rate and found that liver involvement remained significant (*p* = 0.024), aligning with other reports that liver involvement is a significant predictor of poor response rate [57, 67, 84, 86]. However, clinical trial No.42 obtained the opposite conclusion [64]: patients with liver involvement (*n* = 61) who received remestemcel-L had higher DCR (29% versus 5%; *p* = 0.047) and OR (76% vs. 47%, *p* = 0.026) than those who received placebo. The authors attributed the distinctive conclusion to the inaccurate diagnosis of liver involvement, which was based on clinical features, such as elevated transaminase and bilirubin, without histopathological evidence of hepatic lymphocyte infiltration. This so-called liver involvement was probably liver function damage caused by drug toxicity. Therefore, it can be improved by simple symptomatic treatment, leading to the mistaken perception that a response to MSCs is occurring. Whether liver involvement is a good or poor predictor of prognosis needs to be confirmed with future clinical trials with more rigorous diagnostic criteria.

### Diversity in clinical trial design

#### Administration timing

Different MSC administration times appear to have an influence on the efficacy of MSC therapy. In clinical trial No.64, the first MSC treatment was performed within 48 h after the diagnosis of steroid resistance, and significant efficacy was achieved. The 28-day OR was as high as 93%, and the 2-year OS reached 57% [34]. The advantage offered by the early use of MSCs is that patients were not overly pretreated and would have not developed irreversible tissue damage or life-threatening infections. In this trial, MSCs were not only used early, but also used as the only second-line therapy, further demonstrating its efficacy.

However, what would happen if MSCs were infused before steroid resistance develops? Clinical trial No.69 reported using MSCs together with steroid therapy at the onset of aGVHD [74] and trial No.44 used two MSC infusions to treat grades II–IV aGVHD, first given 24–48 h following the diagnosis of GVHD and the second, given three days following the first injection [87]. Both treatments resulted in desirable responses. OR exceeded 90%, notably higher than the OR of MSC treatment for SR GVHD, which was approximately 70% [32, 39, 85, 90]. In conclusion, for GVHD treatment, it is better to use MSCs as soon as possible, preferably before steroid resistance occurs.

There have also been clinical trials evaluating whether infusing MSCs before the onset of GVHD or at the same time of transplantation can prevent GVHD in patients undergoing HSCT, umbilical cord blood (UCB), or peripheral blood stem cell (PBSC) transplantation [43, 91, 92]. Clinical trial No.34 is a pilot study conducted to evaluate whether infusion of MSCs on the same time of PBSC transplantation can prevent GVHD and its impact on OS [93]. The results showed that on the 100th day following transplantation, the incidence of grade II–IV aGVHD in the MSC group was 45%, lower than the 56% of the historical group, especially grade IV aGVHD, which was 10% of the MSC group, lower than the 19% of the historical group. Overall survival at 1 year after transplantation was 80% in the MSC group, significantly higher than the 44% in the historical group (*p* = 0.02). These results indicated that prophylactic application of MSCs resulted in a lower incidence of GVHD, especially severe GVHD and its associated mortality. No.38 is a phase I/II clinical trial conducted to determine whether MSC infusion on the same time of UCB transplantation can reduce the incidence of GVHD. The trial was divided into two groups: MSC infusion with UCB transplantation in MSC group and only UCB transplantation in control group. Comparing the incidence of GVHD in the two groups, the results showed that regardless of acute or chronic GVHD, or grade I–II or grade III–IV aGVHD, no statistical difference existed between the two groups, and the 2-year-OS of the two groups was not significant different, too, suggesting that MSC infusion on the same time of UCB transplantation cannot prevent the occurrence of GVHD [94]. The inconsistent conclusions of the above two trials may be due to the different sources of transplanted cells; the former is PBSC, while the latter is UCB. The influence of the timing of MSC infusion may be related to time-dependent differences in the inflammatory microenvironment in vivo [71]. Therefore, elucidating the mechanism underlying GVHD development will determine the appropriate timing of MSC infusion and elucidate the in vivo effects of transplanted MSCs.

#### Administration dosage

Several studies have compared the efficacy of stratified MSC doses for the prevention and treatment of GVHD [62, 95]. Clinical trial No.14, a multicenter phase II study, was conducted to assess the efficacy of MSCs in the treatment of SR aGVHD. Twenty patients received 1–2 × 10^6^ MSCs/kg, and 13 patients received 3–4 × 10^6^ MSCs/kg. The OR and CR were significantly lower in the former than in the latter (31.6% vs 69.2%; *p* = 0.036 and 15.8% vs 53.8%; *p* = 0.049). Moreover, when OS was evaluated at 1 year, all patients receiving 1–2 × 10^6^ MSCs/kg died, with an OS of 0, significantly lower than the 46.2% of patients receiving 3–4 × 10^6^ MSCs/kg (*p* = 0.012) [96]. The above results illustrate that the OR, CR, and OS of patients with aGVHD to MSCs are positively correlated with the dose of MSCs.

However, these observations are in contrast with other reports that CR or OS did not differ with respect to the dose of MSCs delivered [57, 77, 85]. For example, a phase II, randomized clinical trial (No.44) evaluated the efficacy of MSCs for the treatment of aGVHD. In this trial, MSCs were injected at 8 × 10^6^ MSCs/kg in the high-dose group and 2 × 10^6^ MSCs/kg in the low-dose group; the results showed no difference in the efficacy between low and high MSC doses [87]. This contradiction could be ascribed to the not large enough dose range of MSCs employed in this trial or the small number of patients enrolled [62]. Based on this, investigators designed No.43, a clinical trial with a comparative dose of 2 × 10^6^ MSCs/kg and 10 × 10^6^ MSCs/kg to determine whether differences in the efficacy of aGVHD treatment exist. The dose of MSCs cannot be increased indefinitely: high-dose MSCs may reduce the body’s immune response to infection and increase infectious complications [97]; high numbers of MSCs may lead to embolism or thrombosis [98]. Therefore, future clinical trials should determine the optimum dosage of MSCs that can exert the maximum clinical effects without serious treatment-related adverse events.

#### Administration frequency

In clinical trial No.14, researchers used MSCs once for the treatment of SR aGVHD. Only half of the patients with complete response to MSCs could maintain CR for more than one month, because a single MSC infusion can only play a role in delaying the progression of GVHD [96]. In clinical trial No.44, MSCs were used twice to treat aGVHD. Five patients who had achieved CR had GVHD relapse and required second-line treatment, indicating that MSCs used twice was insufficient to maintain CR [87]. In clinical trial No.42, the 28-day OR was significantly higher in the MSC group than in the placebo group among high-risk aGVHD patients, with no difference between the two groups when evaluating the OS at day 180 [64], indicating that the initial improvement in symptoms did not prolong survival time. OR was evaluated just after completion of the MSC infusion schedule, but OS was evaluated 5 months later than OR. Although patients with PR or mixed responses could receive a weekly MSC infusion for an additional 4 weeks, at least 4 months of MSC-free treatment remained before assessing OS, suggesting that more MSC infusion times are needed to achieve extended survival. Clinical trial No.23 detected the changes in the number and proportion of lymphocyte subsets of patients after each MSC infusion and found that the number of lymphocytes in the responders increased significantly on the 1st and 7th days after each infusion, but not in the non-responders, and the increase was mainly in naïve T/B cells. Moreover, the number of Tregs in responders has also increased significantly, mainly naïve Tregs that can exert strong immunosuppressive functions, while the number of Tregs in non-responders, which were mainly non-Tregs without immunosuppressive function, has not changed. Thus, each MSC infusion is a process of inducing immune tolerance, although the number of naive lymphocytes and Tregs will return to the baseline level before the next infusion, probably due to these regulatory cells leaving the circulatory system and chemotaxis to inflammatory tissues, evading detection in the peripheral blood [75]. Other studies have also used multiple MSC infusions during the treatment period to maintain its efficacy [32, 67].

Studies have also reported results of multiple MSC infusions similar to those obtained using only a single infusion. No.7, a phase I/II clinical trial to evaluate the efficacy of MSCs for aGVHD, included 10 patients, five of whom received 2–4 times of MSCs, but none of them had changed response after receiving the first MSCs. In other words, PR or NR patients would not completely respond due to multiple applications of MSCs [55]. Similarly, clinical trial No.66, which evaluated the efficacy of repeated infusions of MSC in patients with cGVHD, was published as an abstract at the 45th annual meeting of the European Society for Blood and Bone Marrow Transplantation [99]. All patients with cGVHD showed PR after four doses of MSCs. That is, despite repeated infusions of MSCs, no patient achieved CR. To sum up, in GVHD patients who had obtained CR, multiple MSC infusions could maintain the efficacy, but did not improve efficacy in patients who did not obtain CR at the outset.

Overall, the schedule of administration, defined in terms of the most appropriate infusion timing, the best cell dose in each infusion, and the frequency of infusions considerably influenced the results of clinical trials. These findings provide a foundation for investigators hoping to improve the design of future clinical studies for the treatment of GVHD with MSCs.

## Conclusion

With the increasing number of MSC studies showing satisfactory results for prevention or treatment of mouse models of GVHD, the number of clinical trials involving human subjects is also increasing. Previous reviews have summarized clinical trials of MSCs for the prevention or treatment of GVHD, but only 30–40 trials were included in these reviews [100, 101]. To date, however, no comprehensive systematic analysis of clinical trials from multiple databases has been published. In this paper, a total of 79 trials from multiple databases were analyzed. In addition to clinical trials conducted in European and American countries, we also analyzed trials from China, Japan, and Korea, as well as Iran and other Asian countries. This article provides an overview of clinical trials involving the prevention or treatment of GVHD by MSCs, including the study design, duration, geographic location, phase, status, and subtypes of GVHD, to comprehensively evaluate the current status and future development trends of clinical trials for MSC-based prevention or treatment of GVHD worldwide.

Certainly, these clinical trials have some limitations. For example, 72% of clinical trials are open-label, 43% are single-arm, and 23% are non-randomized. Only 85% of the completed clinical trials have reported their results publicly. The reason why other completed trials have not reported their results was unknown. If it was because they were negative, then our statistical analysis of clinical trial results would be missing an important part, and our current positive attitude toward the prevention or treatment of GVHD by MSCs will need serious re-evaluation. However, one statistic is comforting: among the 27 completed clinical trials, 14 have reported follow-up results, of which 7 trials went on for more than 1 year, and the follow-up time of clinical trial No.23 was as long as 6.3 years [75], making it possible to evaluate the long-term safety and efficacy of MSCs.

Among the 79 clinical trials included in this paper, 74 were interventional in nature. Therefore, a comprehensive evaluation and detailed analysis of the results of these trials can help us optimize the usage and dosage of MSCs, find the most suitable patient group, and lay a foundation for the future transformation of MSCs into the clinic. We found that the patients most well-situated to benefit from MSC therapy have these characteristics: (1) younger patients, especially those younger than 10 years old; (2) GVHD with skin but not liver involvement; (3) the degree of tissue damage is mild, manifested as lower levels of TNFRI, IL-2Rα, elafin, CK18 and ST2; (4) a higher proportion of naïve T, B cells and imDCs, better thymic function and lower levels of pro-inflammatory chemokines. Furthermore, MSCs from younger donors, multiple infusions of MSCs, and prompt treatment before SR develops are correlated with maximum clinical benefits (Table 3).

**Table 3:** Characteristics of GVHD patients who are more suitable for MSC therapy

Index	Remarks
Age	Younger than 10 years
GVHD gravity	Skin, but not liver involvement
Degree of tissue damage	Lower levels of TNFRI, IL–2Rα, elafin, CK18, and ST2
Immune tolerance state	Higher proportion of naïve T, B cells and imDCs; Better thymic function; Lower levels of pro-inflammatory chemokines, such as CXCL9, CXCL10, CXCL2, and CCL2
Timing	Preferably before SR occurs
MSCs donor selection	Poor preventive effect of senescent MSCs
Times of MSC administered	Multiple infusions are beneficial to maintain efficacy

In summary, accompanied with the in-depth investigation of the disease-specific biological mechanisms of MSCs and the practice of a large number of clinical trials, it is believed that MSC-based therapy will further contribute to the prophylaxis and treatment of GVHD clinically in future.

## Supplementary Information

**Additional file 1.** List of clinical trials involving MSC prevention or therapy for GVHD..

**Additional file 2.** Characteristics of all selected clinical trials. BM bone marrow, UC umbilical cord, AT adipose tissue, WJ Wharton’s jelly, iPSC induced pluripotent stem cells, N/A not reported.

## Data Availability

The datasets analyzed during the current study are available in the Internet.

## Abbreviations

ALL: Active acute lymphoblastic leukemia
aGVHD: Acute GVHD
AML: Acute myeloid leukemia
ATMSCs: Adipose tissue-derived MSCs
ANZCTR: Australian New Zealand clinical trials registry
BMMSCs: Bone marrow-derived MSCs
ChiCTR: Chinese clinical trial registry
cGVHD: Chronic GVHD
CIBMTR: Center for international blood and marrow transplant research
CRiS: Clinical Research Information Service
CR: Complete response
CK18: Cytokeratin fragment 18
DC: Dendritic cells
DCR: Durable complete response
DEG: Differentially expressed genes
EphB2: Eph receptor B2
FBS: Fetal bovine serum
FGFR1: Fibroblast growth factor receptor 1
FLMSCs: Fetal liver MSCs
GVL: Graft versus leukemia
GVHD: Graft-versus-host disease
HSCT: Hematopoietic stem cell transplantation
ICAM-1: Intercellular adhesion molecule 1
ICTRP: International clinical trials registry platform
IDO: Indoleamine 2,3-dioxygenase
IGF1: Insulin like growth factor 1
IL-1β: Interleukin-1β
IL-2Rα: Interleukin 2 receptor alpha
iPSCs: Induced pluripotent stem cells-derived MSCs
IRCT: Iranian registry of clinical trials
JPRN: Japan primary registries network
MSCs: Mesenchymal stem cells
MM: Multiple myeloma
NR: No response
OR: Overall response
OS: Overall survival
PR: Partial response
PBSC: Peripheral blood stem cell
PD-L1: Programmed cell death 1 ligand 1
PL: Platelet lysate
PPARG: Peroxisome proliferator-activated receptor gamma
Reg3α: Regenerating islet-derived 3α
SOX11: SRY-box transcription factor 11
SR: Steroid-resistance
ST2: Suppression of tumorigenicity 2
TRIM59: Tripartite motif containing 59
TNFR: Tumor necrosis factor receptor
UCB: Umbilical cord blood
UCMSCs: Umbilical cord-derived MSCs
VCAM-1: Vascular cell adhesion molecule 1
WJ: Wharton’s Jelly
