# Supplementary material for: Current status of clinical trials assessing mesenchymal stem cell therapy for graft versus host disease: a systematic review
Source: Stem Cell Res Ther. 2022 Mar 4;13:93. doi: 10.1186/s13287-022-02751-0 (PMC8895864; doi:10.1186/s13287-022-02751-0)
Supplement: Supplementary file 1 — Additional file 1. List of clinical trials involving MSC prevention or therapy for GVHD.. [file 13287_2022_2751_MOESM1_ESM.pdf]

No.	Database	NCT number or ID number	Study title
1	ClinicalTrials.gov	NCT01764100 (EudraCT:2008-007869-23)	Mesenchymal Stromal Cells (MSC) for the Treatment of Severe (Grade II-IV) Steroid-resistant Graft Versus Host Disease (GVHD): a Phase I Trial
2	ClinicalTrials.gov	NCT00827398	Treatment of Steroid Resistant Grade II to IV GVHD by Infusion MSCof Mesenchymal Stem Cells Expanded With Human Plasma and Platelet Lysate a Phase I/II Study
3	ClinicalTrials.gov	NCT02359929	A Phase I Study of Mesenchymal Stromal Cells for the Treatment of Acute and Chronic Graft Versus Host Disease
4	ClinicalTrials.gov	NCT02291770	Treatment of of Chronic Graft-Versus-Host Disease With Mesenchymal Stromal Cells. A Phase III Randomized Open Label Multi-center Study in Southern China.
5	ClinicalTrials.gov	NCT01754454	Open-Label, Single-Center, Self Control, Phase I / II Clinical Trial to Evaluate the Safety and the Efficacy of Umbilical Cord-derived Mesenchymal Stem Cells in Patients With Acute Graft-versus-host Disease
6	ClinicalTrials.gov	NCT02824653	Allogenic Bone Marrow Mesenchymal Stem Cells Infusion in Patients With Steroid-refractory GVHD- A Phase I/II Clinical Trial
7	ClinicalTrials.gov	NCT00447460 (EudraCT:2005-003674-14)	A Phase I/II Trial in Treating Patients With Graft-Versus-Host Disease by the Infusion of Expanded in-Vitro Allogenic Mesenchymal Stem Cell
8	ClinicalTrials.gov	NCT01549665 (KCT0000389)	Umbilical Cord Blood-derived Mesenchymal Stem Cells for the Treatment of Steroid-refractory Acute or Chronic Graft-versus-host-disease
9	ClinicalTrials.gov	NCT02687646	Clinical Trial Phase I / II Graft Versus Host Disease Treatment Refractory to First-line Therapy With Sequential Infusion of Mesenchymal Cells Allogeneic Expanded Adipose Tissue in Vitro
10	ClinicalTrials.gov	NCT00749164	Allogeneic Mesenchymal Stem Cell Infusion for Treatment Of Steroid Resistant GVHD
11	ClinicalTrials.gov	NCT03847844	Cytopeutics® Umbilical Cord Mesenchymal Stem Cells (Cyto-MSC) for Patients With Grade II -IV Acute Graft-Versus-Host Disease: A Phase I/II Clinical Study
12	ClinicalTrials.gov	NCT02379442	A Pilot Study of Early Treatment of Acute Graft Versus Host Disease With Bone Marrow- Derived Mesenchymal Stem Cells and Corticosteroids: Correlation of Disease Severity and Response With Biomarkers
13	ClinicalTrials.gov	NCT00972660	A Phase II, Randomized Study to Evaluate the Safety and Efficacy of Ex-Vivo Cultured Allogenic Mesenchymal Stem Cells For the Treatment of Extensive Chronic Graft Versus Host Disease
14	ClinicalTrials.gov	NCT00603330	Infusion of Mesenchymal Stem Cells as Treatment for Steroid-Resistant Grade II to IV Acute GVHD or Poor Graft Function: a Multicenter Phase II Study
15	ClinicalTrials.gov	NCT04692376	Mesenchymal Stem Cell for Treatment of Chronic Graft-versus-host Disease After Allogenetic Hematopoietic Stem Cell Transplantation
16	ClinicalTrials.gov	NCT02241018	Mesenchymal Stem Cells Combined With CD25 Monoclonal Antibody and Calcineurin Inhibitors for Treatment of Steroid-resistant Acute Graft-

			versus-host Disease After Allogeneic Stem Cell Transplantation
17	ClinicalTrials.gov	NCT02032446	UMBILICAL CORD DERIVED MESENCHYMAL STROMAL CELLS (UC-MSC) FOR THE TREATMENT OF SEVERE (GRADE III-IV) STEROID-RESISTANT GRAFT VERSUS HOST DISEASE (GvHD): A PHASE I/II TRIAL
18	ClinicalTrials.gov	NCT01765634	Mesenchymal Stem Cells From Third-party Donors for Treatment of Refractory Acute Graft-versus-host Disease
19	ClinicalTrials.gov	NCT04738981	Efficacy and Safety of UC-MSCS for the Treatment of Steroid-resistant aGVHD Following Allo-HSCT: A Multicenter, Randomized, Open-label Trial
20	ClinicalTrials.gov	NCT01765660	Mesenchymal Stem Cells From Third-party Donors for Treatment of Refractory Chronic Graft-versus-host Disease
21	ClinicalTrials.gov	NCT01941394	Pilot Study for Safety and Effectiveness Assessment of Bone Marrow Mesenchymal Stem Cell Infusion for Acute Graft-versus-host Disease Prophylaxis and Treatment After Allogenic Bone Marrow Transplantation
22	ClinicalTrials.gov	NCT01526850	Phase Ⅱ/Ⅲ Clinical Trial, Multicenter, Randomized, Controlled, for the Evaluation of Efficacy and Safety of Therapy With Allogenic Mesenchymal Stem Cells in Patients With Chronic Graft Versus Host Disease
23	ClinicalTrials.gov	NCT01522716	Mesenchymal Stromal Cells as Treatment of Chronic Graft-versus-host Disease
24	ClinicalTrials.gov	NCT02770430	A Phase II, Randomized Study to Evaluate the Human Mesenchymal Stem Cells as a First-line Treatment for aGVHD in Patients Steroids Resistant.
25	ClinicalTrials.gov	NCT04744116	A Randomized Controlled Pilot Study of Two Doses of Cord Blood Tissue-Derived Mesenchymal Stromal Cells Combined With Ruxolitinib Versus Ruxolitinib Alone for Therapy of Steroid-Refractory Acute Graft Versus Host Disease
26	ClinicalTrials.gov	NCT01956903	Phase 1/2 Study of Treatment of Refractory Acute Graft-Versus-Host Disease After First Line Therapy by Sequential Infusion of Expanded In-Vitro Allogenic Mesenchymal Stem Cell
27	ClinicalTrials.gov	NCT02336230	A Single-arm, Prospective Study of Remestemcel-L, Ex-vivo Culture-Expanded Adult Human Mesenchymal Stromal Cells, for the Treatment of Pediatric Patients Who Have Failed to Respond to Steroid Treatment for Acute GVHD
28	ClinicalTrials.gov	NCT00314483	Evaluation of Potential Mesenchymal Stem Cells for the Treatment of Graft Versus Host Disease Following an Allogeneic Stem Cell Transplant
29	ClinicalTrials.gov	NCT02055625	Treatment of Oral Mucosa in Patients With Graft-versus-host Disease Following Injection of Mesenchymal Stem Cells - Human Pilot Study
30	ClinicalTrials.gov	NCT02270307	Allogeneic Bone Marrow Transplantation (Allo-BMT) From Human Leukocyte Antigen (HLA) - Identical Related and Unrelated Donors in Patients With Hematological Malignancies With High Risk of Relapse Using Cyclophosphamide (CY) and Mesenchymal Stromal Cells (MSC) as aGVHD
31	ClinicalTrials.gov	NCT04629833 (EudraCT:2019-001462-15)	A Randomised, Open-label, Multicentre, Phase 3 Trial of First-line Treatment With Mesenchymal Stromal Cells MC0518 Versus Best Available Therapy in Adult and Adolescent Subjects With Steroid-refractory Acute

			Graft-versus-host Disease After Allogeneic Haematopoietic Stem Cell Transplantation (IDUNN Trial)
32	ClinicalTrials.gov	NCT01222039 (EudraCT:2008-004014-27)	Multicenter Clinical Trial Phase I/II Randomized, Controlled, for the Evaluation of Safety and Feasibility of Therapy With Two Different Doses of Allogenic Mesenchymal Stem Cells From Adipose Tissue in Patients With Chronic Graft Versus Host Disease.
33	ClinicalTrials.gov	NCT03631589	Treatment of Steroid-resistant Severe Acute Graft-versus-host Disease With Mesenchymal Stem Cells
34	ClinicalTrials.gov	NCT00504803 (EudraCT:2006-004101-26)	Mesenchymal Stem Cell Infusion as Prevention for Graft Rejection and Graft-versus-host Disease After Allogeneic Hematopoietic Cell Transplantation With Nonmyeloablative Conditioning: a Pilot Study
35	ClinicalTrials.gov	NCT02923375	An Open-Label Phase 1 Study to Investigate the Safety and Efficacy of CYP-001 for the Treatment of Adults With Steroid-Resistant Acute Graft Versus Host Disease
36	ClinicalTrials.gov	NCT03123458	Clonal Fetal Mesenchymal Stem Cells (cfMSCs) for the Control of Immune-related Disorders
37	ClinicalTrials.gov	NCT04328714	Interferon γ-Primed Mesenchymal Stromal Cells as Prophylaxis for Acute Graft v Host Disease After Allogeneic Hematopoietic Cell Transplantation for Patients With Hematologic Malignancies and Myelodysplasia
38	ClinicalTrials.gov	NCT00823316	Phase 1/2 Study of Umbilical Cord Blood-Drived Mesenchymal Stem Cells Infusion for Promotion of Engraftment and Prevention of an Graft Rejection and Graft-versus-Host Disease After Unrelated Hematopoietic Stem Cell Transplantation.
39	ClinicalTrials.gov	NCT01045382	Co-transplantation of Mesenchymal Stem Cells and HLA-mismatched Allogeneic Hematopoietic Cells After Nonmyeloablative Conditioning: a Phase II Randomized Double-blind Study
40	ClinicalTrials.gov	NCT00361049	Donor Mesenchymal Stem Cell Infusion for Treatment of Graft Versus Host Disease: A Phase I Trial
41	ClinicalTrials.gov	NCT03106662	Mesenchymal Stem Cell Infusion in Haploidentical Hematopoietic Stem Cell Transplantation in Patients With Hematological Malignancies
42	ClinicalTrials.gov	NCT00366145	A Phase III, Randomized, Double Blind, Placebo-Controlled Study to Evaluate the Efficacy and Safety of Prochymal™ (Ex-vivo Cultured Adult Human Mesenchymal Stem Cells) Infusion for the Treatment of Patients Who Have Failed to Respond to Steroid Treatment for Acute GVHD
43	ClinicalTrials.gov	NCT03158896	A Phase I Study To Evaluate the Safety of Umbilical Cord - Derived, Ex-Vivo Cultured and Expanded Wharton's Jelly Mesenchymal Stem Cells for the Treatment of De Novo High Risk Acute or Steroid Refractory Acute Graft Versus Host Disease
44	ClinicalTrials.gov	NCT00136903	A Phase II, Randomized Study to Evaluate the Safety and Efficacy of Prochymal (Ex-vivo Cultured Adult Human Mesenchymal Stem Cells) For the Treatment of Acute GVHD in Patients Who Receive Allogeneic Hematopoietic Stem Cell Transplantation

45	ClinicalTrials.gov	NCT01318330	Five-Week, Multi-center, Phase I Clinical Trial to Evaluate Safety of Homeo-GH After Intra Venus Administration for the Treatment of Graft Versus Host Disease Patients
46	ClinicalTrials.gov	NCT00284986	A Phase II Open Label Study to Evaluate the Safety and Efficacy of Prochymal (Ex-vivo Cultured Adult Human Mesenchymal Stem Cells) Infusion for the Salvage of Treatment-Refractory Acute GVHD Patients
47	ClinicalTrials.gov	NCT01589549	A Phase 2 Trial of Standard of Care Treatment Versus Mesenchymal Stromal Cell Therapy Together With Standard of Care for the Treatment of de Novo Acute Graft Versus Host Disease Following Allogeneic Bone Marrow Transplantation
48	ClinicalTrials.gov	NCT04189432 (KCT0003656)	A Multicenter, Randomized, Parallel Group, Double-blind, Phase 2 Trial to Evaluate Efficacy and Safety of SCM-CGH in Patients With Steroid-Refractory or Dependent Chronic Graft-Versus-Host Disease
49	ClinicalTrials.gov	NCT00562497	A Phase III, Randomized, Double-Blind, Placebo-Controlled Study to Evaluate the Efficacy and Safety of Prochymal™ Infusion in Combination With Corticosteroids for the Treatment of Newly Diagnosed Acute GVHD
50	ClinicalTrials.gov	NCT00759018	Expanded Access of Prochymal (Ex-vivo Cultured Adult Human Mesenchymal Stem Cells) Infusion for the Treatment of Pediatric Patients Who Have Failed to Respond to Steroid Treatment for Acute GVHD
51	ClinicalTrials.gov	NCT00826046	Prochymal® Expanded Access for Adults Who Have Failed Steroid Treatment for Acute Graft Versus Host Disease (GVHD)
52	ClinicalTrials.gov	NCT00081055	OTI-010 for Graft-Versus-Host Disease Prophylaxis in Treating Patients Who Are Undergoing Donor Peripheral Stem Cell Transplantation for Hematologic Malignancies
53	ClinicalTrials.gov	NCT02172937	Decidual Stromal Cells as Treatment for Acute Graft Versus Host Disease, a Phase 1-2 Study
54	ChiCTR	ChiCTR2000039821	Clinical Research Protocol of Human Amniotic Membrane Epithelial Stem Cells for the Prevent of Acute Graft Versus Host Disease After Cord Blood Stem Cell Transplantation
55	ChiCTR	ChiCTR2000035740	To evaluate the therapeutic effect of HUC-MSC PLEB001 in steroid-resistant acute graft versus host disease (aGVHD)： a multicenter randomised, double-blind, placebo-controlled, phase 2 trial
56	ChiCTR	ChiCTR1900022292	Clinical research of Umbilical Cord–Derived Mesenchymal Stromal Cells in the Prophylaxis of Graft-Versus-Host Disease After HLA-Haploidentical Stem-Cell Transplantation， a multicenter， randomized， controlled and open trial
57	ChiCTR	ChiCTR-ONC-17011480	Clincal study of umbilical cord-derived mesenchymal stem cells in the graft-versus-host disease after allogeneic hematopoietic stem cell transplantation
58	ChiCTR	ChiCTR-IIR-16007806	Clincal study of umbilical cord-derived mesenchymal stem cells in the prophylaxis of chronic graft-versus-host disease after HLA-haploidentical stem cell transpantation
59	ChiCTR	ChiCTR-INR-16008399	Cotransplantation of haploidentical peripheral blood stem cells and mesenchymal stem cells for acute leukemia: a randomized, controlled

			clinical study
60	ChiCTR	ChiCTR-IOR-15006330	Clinical application of Umbilical Cord-Derived Mesenchymal Stem Cells in the Prophylaxis of Chronic Graft-versus-Host Disease after HLA-haploidentical stem cell transplantation-A Prospective, Randomized, Parallel-group Study
61	JPRN	JPRN-UMIN000029945	Phase I/II trial of AM01 (amnion-derived mesenchymal stem cells) for steroid-refractory acute graft-versus-host disease (GVHD) after allogeneic hematopoietic stem cell transplantation
62	JPRN	JPRN-UMINC000000423	Efficacy of mesenchymal stem cells for treatment of refractory acute GVHD after stem cell transplantation
63	JPRN	JPRN-UMIN000015017	Amniotic membrane-derived mesenchymal stromal cells for the treatment of steroid-resistant acute GVHD
64	JPRN	JPRN-UMIN000001716	A Phase I/II study of JR-031 for the patients of standard primary treatment-refractory acute graft-versus-host disease (acute GVHD) after hematopoietic stem cell transplantation
65	JPRN	JPRN-UMIN000006719	A Phase II/III trial of JR-031 for the treatment of steroid-refractory acute Graft-versus-Host disease after hematopoietic stem cell transplantion
66	CRiS	KCT0001894	A phase I study to evaluate the safety of four repeated infusions of bone marrow-derived mesenchymal stem cells for the treatment of steroid-refractory and progressive chronic graft-versus host disease.
67	IRCT	IRCT2014072618603N1	Adult Human Mesenchymal Stem Cells for the treatment of Steroid-Refractory Acute Graft-versus-Host Disease
68	ANZCTR	ACTRN12608000129381	Cotransplantation of mesenchymal stem cells with nonmyeloablative haploidentical peripheral blood stem cells without T cells deleted for high-risk acute leukemia: to reduce the severity of graf versus host disease and relapse.
69	ANZCTR	ACTRN12610000068066	A phase 1 study to assess the safety of mesenchymal stromal cells to treat steroid-refractory graft versus host disease after allogeneic haemopoietic stem cell tranplantation.
70	ANZCTR	ACTRN12607000430437	A phase 1 multicentre open label dose-escalation study of unrelated, major histocompatibility (MHC)-unmatched mesenchymals stem cells (MSC) for the treatment of steroid refractory acute graft versus host disease in recipients of allogeneic heamatopoietic stem cell transplants
71	EU Clinical Trials Register	EudraCT:2006-004420-37	A Phase III, Randomized, Double Blind, Placebo-Controlled Study to Evaluate the Efficacy and Safety of Prochymal (Ex-vivo Cultured Adult Human Mesenchymal Stem Cells) Infusion for the Treatment of Steroid Refractory Acute GVHD Patients
72	EU Clinical Trials Register	EudraCT:2007-004310-14	Infusion of mesenchymal stem cells as treatment for steroid resistant grade II to IV acute GVHD or poor graft function: a multicenter phase II study

73	EU Clinical Trials Register	EudraCT:2011-003237-33	Treatment of severe GVHD after allogeneic hematopoietic stem cell transplanatation with MSCs and steroid versus steroids alone.A prospective double-blind placebo-controlled randomized phase III trial
74	EU Clinical Trials Register	EudraCT:2012-004915-30	Treatment of severe steroid-refractory acute GvHD with mesenchymal stromal cells. A phase III, randomized double-blind multi-center HOVON study.
75	EU Clinical Trials Register	EudraCT:2013-003626-88	The utilization of mesenchymal stem cells (MSC) for the treatment of graft versus host disease (GVHD) after allogeneic stem cell transplantation.
76	EU Clinical Trials Register	EudraCT:2009-011817-26	A pilot study to assess the feasibility of unrelated umbilical cord blood transplantation with coinfusion of third-party mesenchymal stem cells after myeloablative or nonmyeloablative conditioning in adult patients with hematological malignancies.
77	EU Clinical Trials Register	EudraCT:2009-014980-38	Co-transplantation of mesenchymal stem cells and HLA-mismatched allogeneic hematopoietic cells after reduced-intensity conditioning: a phase II randomized double-blind study.
78	EU Clinical Trials Register	EudraCT:2008-005594-35	Randomized double-blind study of mesenchymal stem cells (MSC) in patients undergoing matched unrelated allogeneic bone marrow or peripheral blood stem cell transplantation- A European multicentre study.
79	EU Clinical Trials Register	EudraCT:2007-003341-32	Treatment of steroid resistant grade II to IV acute GVHD by infusion of mesenchymal stem cells expanded with human plasma and platelet lysate; a phase I/II study
