# Supplementary material for: Current status of clinical trials assessing mesenchymal stem cell therapy for graft versus host disease: a systematic review
Source: Stem Cell Res Ther. 2022 Mar 4;13:93. doi: 10.1186/s13287-022-02751-0 (PMC8895864; doi:10.1186/s13287-022-02751-0)
Supplement: Supplementary file 2 — Additional file 2. Characteristics of all selected clinical trials. BM bone marrow, UC umbilical cord, AT adipose tissue, WJ Wharton’s jelly, iPSC induced pluripotent stem cells, N/A not reported. [file 13287_2022_2751_MOESM2_ESM.pdf]

| Clinical trial | Phase | Masking | Allocation | Intervention Model | Study Type | Status | Number Enrolled | Composition | Study Start | Locations | Research Center | Origin | Source | Objective | Subcategory of GVHD |
|---|---|---|---|---|---|---|---|---|---|---|---|---|---|---|---|
| No.1 | 1 | Open Lable | N/A | Singe Group Assignment | Interventional | Unknown | 10 | Child, Adult, Older adult | 2009 | Italy | Multi-center | Allogeneic | BM | Treatment | Both |
| No.2 | 1+2 | Open Lable | N/A | Singe Group Assignment | Interventional | Completed | 50 | Child, Adult, Older adult | 2009 | Netherlands | Multi-center | Allogeneic | BM | Treatment | Both |
| No.3 | 1 | Open Lable | Non-randomized | Parallel Assignment | Interventional | Completed | 11 | Child, Adult, Older adult | 2015 | America | One-center | Autologous | BM | Treatment | Both |
| No.4 | 3 | Open Lable | Randomized | Parallel Assignment | Interventional | Unknown | 130 | Child, Adult, Older adult | 2014 | China | Multi-center | N/A | N/A | Treatment | Chronic |
| No.5 | 1+2 | Open Lable | N/A | Singe Group Assignment | Interventional | Unknown | 30 | Adult,Older adult | 2012 | China | Multi-center | N/A | UC | Treatment | Acute |
| No.6 | 1+2 | Open Lable | N/A | Singe Group Assignment | Interventional | Completed | 10 | Child, Adult, Older adult | 2015 | Pakistan | One-center | Allogeneic | BM | Treatment | Acute |
| No.7 | 1+2 | Open Lable | Non-randomized | Singe Group Assignment | Interventional | Unknown | 15 | Adult,Older adult | 2007 | Spain | Multi-center | Allogeneic | BM | Treatment | Both |
| No.8 | 1+2 | Open Lable | N/A | Singe Group Assignment | Interventional | Unknown | 30 | Child, Adult | 2012 | Korea | One-center | Allogeneic | UC | Treatment | Both |
| No.9 | 1+2 | Open Lable | N/A | Singe Group Assignment | Interventional | Not Recruiting | 16 | Adult,Older adult | 2016 | Spain | Multi-center | Allogeneic | AT | Treatment | Acute |
| No.10 | 1+2 | Open Lable | Non-randomized | Singe Group Assignment | Interventional | Unknown | 20 | Child, Adult, Older adult | 2009 | Israel | One-center | Allogeneic | UC | Treatment | Acute |
| No.11 | 1+2 | Triple blind | Randomized | Parallel Assignment | Interventional | Recruiting | 40 | Child, Adult, Older adult | 2019 | Malaysia | One-center | Allogeneic | UC | Treatment | Acute |
| No.12 | 1+2 | Open Lable | N/A | Singe Group Assignment | Interventional | Terminated | 1 | Child | 2015 | America | One-center | Allogeneic | BM | Treatment | Acute |
| No.13 | 2 | Open Lable | Randomized | Parallel Assignment | Interventional | Unknown | 52 | Child, Adult, Older adult | 2009 | China | One-center | Allogeneic | BM | Treatment | Chronic |
| No.14 | 2 | Open Lable | Non-randomized | Singe Group Assignment | Interventional | Recruiting | 100 | Child, Adult, Older adult | 2008 | Belgium, Netherlands | Multi-center | Allogeneic | BM | Treatment | Acute |
| No.15 | 2 | Open Lable | Randomized | Parallel Assignment | Interventional | Recruiting | 152 | Adult,Older adult | 2021 | China | One-center | N/A | N/A | Treatment | Chronic |
| No.16 | 2+3 | Open Lable | Randomized | Parallel Assignment | Interventional | Unknown | 200 | Child, Adult, Older adult | 2014 | China | One-center | Allogeneic | BM | Treatment | Acute |
| No.17 | 1+2 | Open Lable | N/A | Singe Group Assignment | Interventional | Unknown | 47 | Child, Adult, Older adult | 2013 | Italy | Multi-center | N/A | UC | Treatment | Acute |
| No.18 | 2 | Open Lable | Non-randomized | Parallel Assignment | Interventional | Unknown | 40 | Child, Adult, Older adult | 2013 | China | One-center | Allogeneic | BM | Treatment | Acute |
| No.19 | 3 | Open Lable | Randomized | Parallel Assignment | Interventional | Not Recruiting | 130 | Adult,Older adult | 2021 | China | One-center | N/A | UC | Treatment | Acute |
| No.20 | 2 | Open Lable | Non-randomized | Parallel Assignment | Interventional | Unknown | 60 | Child, Adult, Older adult | 2013 | China | One-center | Allogeneic | BM | Treatment | Chronic |
| No.21 | 2 | Open Lable | Randomized | Parallel Assignment | Interventional | Unknown | 70 | Adult | 2007 | Russia | One-center | Allogeneic | BM | Prevention | Acute |
| No.22 | 2+3 | Open Lable | Randomized | Parallel Assignment | Interventional | Unknown | 100 | Child, Adult | 2012 | China | Multi-center | Allogeneic | N/A | Treatment | Chronic |
| No.23 | 1 | Open Lable | N/A | Singe Group Assignment | Interventional | Terminated | 11 | Adult,Older adult | 2011 | Sweden | One-center | Allogeneic | BM | Treatment | Chronic |
| No.24 | 2 | Open Lable | Randomized | Crossover Assignment | Interventional | Unknown | 90 | Child, Adult, Older adult | 2015 | Brazil | One-center | N/A | BM | Treatment | Acute |
| No.25 | 1 | Open Lable | Randomized | Parallel Assignment | Interventional | Recruiting | 24 | Child, Adult, Older adult | 2021 | America | One-center | N/A | UC | Treatment | Acute |
| No.26 | 1+2 | Open Lable | N/A | Singe Group Assignment | Interventional | Completed | 15 | Adult,Older adult | 2010 | Spain | One-center | Allogeneic | N/A | Treatment | Acute |
| No.27 | 3 | Open Lable | N/A | Singe Group Assignment | Interventional | Completed | 55 | Child | 2015 | America | Multi-center | Allogeneic | BM | Treatment | Acute |
| No.28 | 1+2 | Open Lable | Non-randomized | Singe Group Assignment | Interventional | Unknown | 25 | Child, Adult | 2007 | India | One-center | Allogeneic | N/A | Treatment | Acute |
| No.29 | 1+2 | Open Lable | N/A | Singe Group Assignment | Interventional | Withdrawn | 0 | Adult,Older adult | 2014 | Sweden | One-center | Allogeneic | N/A | Treatment | Chronic |
| No.30 | 2+3 | Open Lable | N/A | Singe Group Assignment | Interventional | Unknown | 40 | Adult,Older adult | 2014 | Russia | One-center | N/A | N/A | Prevention | Acute |

| | | | | | | | | | | | | | | | |
|---|---|---|---|---|---|---|---|---|---|---|---|---|---|---|---|
| No.31 | 3 | Open Lable | Randomized | Parallel Assignment | Interventional | Recruiting | 210 | Child, Adult, Older adult | 2021 | France, Germany, Poland, Spain | Multi-center | Allogeneic | BM | Treatment | Acute |
| No.32 | 1+2 | Open Lable | Randomized | Parallel Assignment | Interventional | Completed | 19 | Adult,Older adult | 2010 | Spain | Multi-center | Allogeneic | AT | Treatment | Chronic |
| No.33 | 2+3 | Open Lable | N/A | Singe Group Assignment | Interventional | Recruiting | 50 | Child, Adult, Older adult | 2017 | China | One-center | N/A | N/A | Treatment | Acute |
| No.34 | 2 | Open Lable | Non-randomized | Singe Group Assignment | Interventional | Completed | 30 | Child, Adult, Older adult | 2006 | Belgium | One-center | Allogeneic | BM | Prevention | Both |
| No.35 | 1 | Open Lable | Non-randomized | Sequential Assignment | Interventional | Completed | 16 | Adult,Older adult | 2017 | Australia, England | Multi-center | Allogeneic | iPSC | Treatment | Acute |
| No.36 | 1 | Open Lable | N/A | Singe Group Assignment | Interventional | Unknown | 100 | Child, Adult, Older adult | 2016 | China | Multi-center | Allogeneic | Fetus | Treatment | Both |
| No.37 | 1 | Open Lable | Non-randomized | Sequential Assignment | Interventional | Not Recruiting | 45 | Child, Adult, Older adult | 2021 | America | Multi-center | Allogeneic | BM | Prevention | Acute |
| No.38 | 1+2 | Open Lable | Randomized | Parallel Assignment | Interventional | Completed | 10 | Child, Adult | 2008 | Korea | One-center | Allogeneic | UC | Prevention | N/A |
| No.39 | 2 | Double blind | Randomized | Parallel Assignment | Interventional | Terminated | 39 | Child, Adult, Older adult | 2010 | Belgium | Multi-center | Allogeneic | N/A | Prevention | Both |
| No.40 | 1 | Open Lable | N/A | Singe Group Assignment | Interventional | Completed | 49 | Adult,Older adult | 2004 | America | Multi-center | Allogeneic | N/A | Treatment | Both |
| No.41 | 3 | Open Lable | N/A | Singe Group Assignment | Interventional | Completed | 6 | Adult,Older adult | 2014 | Turkey | One-center | Allogeneic | BM | Prevention | N/A |
| No.42 | 3 | Quadruple blind | Randomized | Parallel Assignment | Interventional | Completed | 260 | Child, Adult, Older adult | 2006 | America, Australia, Canada, Italy,Germany, Spain, England | Multi-center | Allogeneic | BM | Treatment | Acute |
| No.43 | 1 | Open Lable | N/A | Sequential Assignment | Interventional | Completed | 10 | Adult,Older adult | 2018 | America | One-center | Allogeneic | WJ | Treatment | Acute |
| No.44 | 2 | Open Lable | Randomized | Parallel Assignment | Interventional | Completed | 33 | Adult,Older adult | 2005 | America | Multi-center | Allogeneic | BM | Treatment | Acute |
| No.45 | 1 | Open Lable | N/A | Singe Group Assignment | Interventional | Completed | 10 | Adult,Older adult | 2010 | Korea | Multi-center | Allogeneic | BM | Treatment | Both |
| No.46 | 2 | Open Lable | N/A | Singe Group Assignment | Interventional | Completed | 15 | Child, Adult, Older adult | 2005 | America | One-center | Allogeneic | BM | Treatment | Acute |
| No.47 | 2 | Open Lable | Randomized | Parallel Assignment | Interventional | Unknown | 66 | Adult | 2012 | Australia | One-center | Allogeneic | N/A | Treatment | Acute |
| No.48 | 2 | Triple blind | Randomized | Parallel Assignment | Interventional | Recruiting | 77 | Adult,Older adult | 2016 | Korea | Multi-center | Allogeneic | BM | Treatment | Chronic |
| No.49 | 3 | Triple blind | Randomized | Parallel Assignment | Interventional | Completed | 192 | Adult,Older adult | 2008 | America, Australia, Canada | Multi-center | Allogeneic | BM | Treatment | Acute |
| No.50 | N/A | N/A | N/A | N/A | Expanded Access | No Longer Available | N/A | Child | 2007 | America, Canada, England, Italy, Finland, Spain, New Zealand, Australia | Multi-center | Allogeneic | BM | Treatment | Acute |
| No.51 | N/A | N/A | N/A | N/A | Expanded Access | No Longer Available | N/A | Adult,Older adult | N/A | America | Multi-center | Allogeneic | BM | Treatment | Acute |
| No.52 | 2 | Double blind | Randomized | N/A | Interventional | Withdrawn | 0 | Adult | N/A | America | Multi-center | Autologous | N/A | Prevention | N/A |
| No.53 | 1+2 | Open Lable | Non-randomized | Singe Group Assignment | Interventional | Unknown | 50 | Child, Adult, Older adult | 2011 | Sweden | One-center | Allogeneic | Placenta | Treatment | Acute |
| No.54 | 1 | N/A | Non-randomized | Singe Group Assignment | Interventional | Recruiting | 12 | Child | 2021 | China | One-center | N/A | Amnion | Prevention | Acute |
| No.55 | 2 | Double blind | Randomized | Parallel Assignment | Interventional | Not Recruiting | 96 | Child, Adult, Older adult | 2020 | China | Multi-center | N/A | UC | Treatment | Acute |
| No.56 | 2/3 | Open Lable | Randomized | Parallel Assignment | Interventional | Recruiting | 192 | Adult | 2019 | China | Multi-center | N/A | UC | Prevention | Both |
| No.57 | N/A | N/A | Randomized | Singe Group Assignment | Observational | Recruiting | 30 | Child, Adult, Older adult | 2017 | China | One-center | N/A | UC | Treatment | N/A |
| No.58 | 2 | Double blind | Randomized | Parallel Assignment | Interventional | Not Recruiting | 156 | Adult | 2016 | China | Multi-center | N/A | UC | Prevention | Chronic |
| No.59 | N/A | N/A | Randomized | Parallel Assignment | Interventional | Recruiting | 36 | Child, Adult | 2016 | China | One-center | N/A | N/A | Prevention | Both |
| No.60 | 2 | Double blind | Randomized | Parallel Assignment | Interventional | Not Recruiting | 120 | Adult | 2015 | China | Multi-center | Allogeneic | UC | Prevention | Chronic |
| No.61 | 1+2 | Open Lable | Non-randomized | Singe Group Assignment | Interventional | Not Recruiting | 12 | Child, Adult, Older adult | 2017 | Japan | Multi-center | N/A | Amnion | Treatment | Acute |

| | | | | | | | | | | | | | | | |
|---|---|---|---|---|---|---|---|---|---|---|---|---|---|---|---|
| No.62 | N/A | Open Lable | Non-randomized | Singe Group Assignment | Interventional | Completed | 5 | Adult | 2006 | Japan | N/A | N/A | N/A | Treatment | Acute |
| No.63 | 1 | Open Lable | Non-randomized | Singe Group Assignment | Interventional | Completed | 5 | Child, Adult, Older adult | 2014 | Japan | N/A | N/A | Amnion | Treatment | Acute |
| No.64 | 1+2 | Open Lable | Non-randomized | Singe Group Assignment | Interventional | Completed | 14 | Child, Adult, Older adult | 2009 | Japan | Multi-center | Allogeneic | BM | Treatment | Acute |
| No.65 | 2+3 | Open Lable | Non-randomized | Singe Group Assignment | Interventional | Completed | 25 | Child, Adult, Older adult | 2011 | Japan | N/A | Allogeneic | BM | Treatment | Acute |
| No.66 | 1 | Open Lable | N/A | Singe Group Assignment | Interventional | Completed | 10 | Child, Adult, Older adult | 2017 | Korea | One-center | Allogeneic | BM | Treatment | Chronic |
| No.67 | 3 | Open Lable | Randomized | Parallel Assignment | Interventional | Unknown | 20 | Child, Adult | 2016 | Iran | One-center | Allogeneic | N/A | Treatment | Acute |
| No.68 | 2 | Open Lable | Randomized | Parallel Assignment | Interventional | Recruiting | 40 | Child, Adult | 2008 | China | Multi-center | N/A | N/A | Prevention | Both |
| No.69 | 1 | N/A | N/A | N/A | N/A | Completed | 19 | Adult | 2007 | Australia | One-center | Allogeneic | BM | Treatment | Both |
| No.70 | 1 | Open Lable | N/A | N/A | N/A | Not Recruiting | N/A | Adult | 2007 | Australia | Multi-center | Allogeneic | BM | Treatment | Acute |
| No.71 | 3 | Double blind | Randomized | N/A | Interventional | Completed | 240 | Child, Adult | 2007 | Italy, England, Spain | Multi-center | Allogeneic | BM | Treatment | Acute |
| No.72 | 2 | Open Lable | Non-randomized | Singe Group Assignment | Interventional | Ongoing | 120 | Child, Adult, Older adult | 2007 | Belgium | Multi-center | N/A | N/A | Treatment | Acute |
| No.73 | 3 | Double blind | Randomized | Parallel Assignment | Interventional | Ongoing | 200 | Child, Adult, Older adult | 2013 | Netherlands | Multi-center | Allogeneic | BM | Treatment | Acute |
| No.74 | 3 | Double blind | Randomized | Parallel Assignment | Interventional | Ongoing | 150 | Child, Adult, Older adult | 2013 | Netherlands, Germany, Belgium, Spain | Multi-center | N/A | BM | Treatment | Acute |
| No.75 | 2 | Open Lable | N/A | N/A | Interventional | Completed | 25 | Adult,Older adult | 2014 | Czech | One-center | Both | N/A | Treatment | Both |
| No.76 | 2 | N/A | N/A | N/A | Interventional | Completed | 20 | Child, Adult | 2010 | Belgium | Multi-center | Allogeneic | N/A | Prevention | Both |
| No.77 | 2 | Double blind | Randomized | Parallel Assignment | Interventional | Ongoing | 120 | Child, Adult, Older adult | 2010 | Belgium | Multi-center | Allogeneic | N/A | Prevention | Both |
| No.78 | 3 | Double blind | Randomized | N/A | Interventional | Ongoing | 172 | Child, Adult, Older adult | 2009 | Belgium | Multi-center | Both | N/A | Prevention | Acute |
| No.79 | 1+2 | N/A | N/A | N/A | Interventional | Completed | 10 | Child, Adult | 2008 | Netherlands | One-center | N/A | N/A | Treatment | Acute |

BM represents bone marrow;UC represents umbilical cord;AT represents adipose tissue;iPSC represents induced pluripotent stem cells;WJ represents Wharton's jelly;N/A represents not reported
